# A role for the poly-asparagine repeat in the *Plasmodium* histone acetyltransferase, PfGCN5

**DOI:** 10.1038/s41467-026-74632-6

**Published:** 2026-06-20

**Authors:** Kelly Rubiano, Aaron Morris, Francisca D. L. Vitorino, Jasper Travis, Benjamin A. Garcia, Sumit Mukherjee, Daniel E. Goldberg

**Affiliations:** 1 Department of Medicine, Division of Infectious Diseases, Washington University School of Medicine, St. Louis, MO USA; 2 Department of Molecular Microbiology, Washington University School of Medicine, St. Louis, MO USA; 3 Department of Biology, Washington University in St. Louis, St. Louis, MO USA; 4 Department of Biochemistry and Molecular Biophysics, Washington University School of Medicine, St. Louis, MO USA; 5 Department of Biology, Texas State University, San Marcos, TX USA

**Keywords:** Parasite biology, Parasite evolution

## Abstract

*Plasmodium falciparum* possesses one of the most AT-rich genomes in nature (80.6%). A consequence is an asparagine-rich proteome. A quarter of *P. falciparum* proteins possess poly-asparagine repeats that can extend more than 100 residues. The role of these repeats has remained a mystery in the biology of this parasite. Here, we report that the poly-asparagine repeat-containing Nterminus of the histone acetyltransferase PfGCN5 associates with the C-terminal catalytic domain after cleavage in the nucleus. Deletion of the repeat destabilizes the N-terminal polypeptide, leading to impaired parasite development and growth, particularly under stress conditions. Using high-resolution mass spectrometry and western blotting analysis, we uncovered a profound effect of the poly-asparagine repeat on acetylation of histones H3, H3.3, and H4. These findings suggest that the poly-asparagine repeat contributes to PfGCN5 acetyltransferase activity, a role previously attributed solely to its C-terminal domain. This report of a function for a poly-asparagine repeat in *P. falciparum* expands our understanding of a pervasive characteristic of its proteome.

## Abstract (web summary)

We find that *Plasmodium falciparum* GCN5 has a poly-Asn repeat crucial for N-terminus association with the catalytic C-terminus, affecting histone acetylation, cell growth and stress survival. This provides a function for a poly-Asn region, found in >1000 parasite proteins.

## Introduction

The human malaria parasite, *Plasmodium falciparum*, exhibits one of the most AT-rich genomes (80.6%) sequenced to date, and among the *Plasmodium* species infecting humans, it outruns its peers^1–3^. As expected, amino acid residues with high AT content, such as asparagine (AAT/AAC) and lysine (AAA/AAG), are enriched in the *P. falciparum* proteome^4^. However, codon bias analyses established a preference for asparagine over lysine residues despite equivalent AT-richness (83.3%)^4,5^. Remarkably, asparagine (N) residues are found in tandem predominantly in intragenic regions, forming low-complexity, intrinsically disordered regions in about 24% of the *P. falciparum* proteome, when considering at least 30 N in an 80-residue window^4^. *P. falciparum’s* poly-N repeats are either perfect or imperfect. When imperfect, they are typically interrupted by polar residues, such as serine, aspartic acid, and glutamic acid, as well as the aromatic residue tyrosine. These repeats average 37 residues but can exceed 100 residues in length^6^. Since poly-N repeats in *P. falciparum* proteins do not cluster in specific protein families, metabolic pathways, or parasite stages, the functional contribution of these repeats has been elusive^7^. Structural mapping of poly-N repeats suggests that they are unlikely to adopt globular folds due to their flexible and hydrophilic nature, which favors protrusion from protein cores^4^. This raises the possibility that poly-N repeats enhance protein function, adopt new functions, or confer protein stability without interfering with basal protein activity. Furthermore, poly-N repeats exhibit a reversible aggregation propensity that increases with length and temperature^8,9^. Thus, the evolutionary forces shaping the *P. falciparum* proteome, an organism that withstands febrile cycles, are of interest.

To date, only two *P. falciparum* proteins containing poly-N repeats have been experimentally characterized. In the first case, the parasite chaperone PfHsp110, but not its human or yeast counterparts, efficiently prevented the heat shock (HS)-induced aggregation of the parasite’s putative CDK2-regulatory subunit, which contains 83 consecutive N residues, highlighting the robust specialization that parasite chaperones have evolved^10^. In the second case, removal of the 28-residue poly-N tract from the essential proteasome clamp subunit, Rpn6, had no effect on protein expression, function, or parasite viability at 37 °C or 41 °C, suggesting that some poly-N repeats may lack functional properties^11^. Given the large group of poly-N repeat-containing proteins that remain uncharacterized in *P. falciparum*, we sought to investigate the role of an imperfect repeat of 98 residues containing 81 N, located in the N-terminus of the parasite’s histone acetyltransferase GCN5 (PfGCN5). GCN5 is highly conserved among eukaryotes with two well-characterized C-terminal domains: a histone acetyltransferase (HAT) domain, which catalyzes the transfer of acetyl groups to lysine residues, and a bromodomain (BrD), which binds acetylated substrates to support enzymatic processivity^12^. Furthermore, GCN5 participates in the megadalton SAGA complex, where, together with ADA2, ADA3, and SGF29, it forms the HAT catalytic module of the complex^13,14^. In *P. falciparum*, GCN5 is essential and regulates the expression of a subset of genes involved in development, invasion, and stress response^15–18^. Seminal work on PfGCN5 has underscored its pivotal role in catalyzing histone acetylation marks associated with euchromatin^17,19,20^. Deletion of the BrD from PfGCN5 leads to decreased histone acetylation and altered chromatin architecture, resulting in defective intraerythrocytic development and dysregulated sexual commitment^17,18^. BrD deletion lines also exhibit impaired maturation of gametocytes and mosquito stages^18^. Therefore, PfGCN5 holds a critical role in modifying the chromatin landscape across the parasite’s life cycle.

Our findings show that the N-terminal fragment containing the poly-N repeat in PfGCN5 contributes to histone acetylation by associating with the C-terminal catalytic domain. We characterized a poly-N repeat deletion line and found impaired parasite growth, particularly under stress conditions. We explored the nucleocytoplasmic live-cell dynamics of the N-terminal polypeptide and determined that it is destabilized when the poly-N repeat is removed. Furthermore, we have expanded the known repertoire of acetylation marks catalyzed by PfGCN5’s activity to H3.3 and H4.

## Results

### The N-terminus of PfGCN5 containing the poly-asparagine repeat is important for parasite growth

**Fig. 1:**
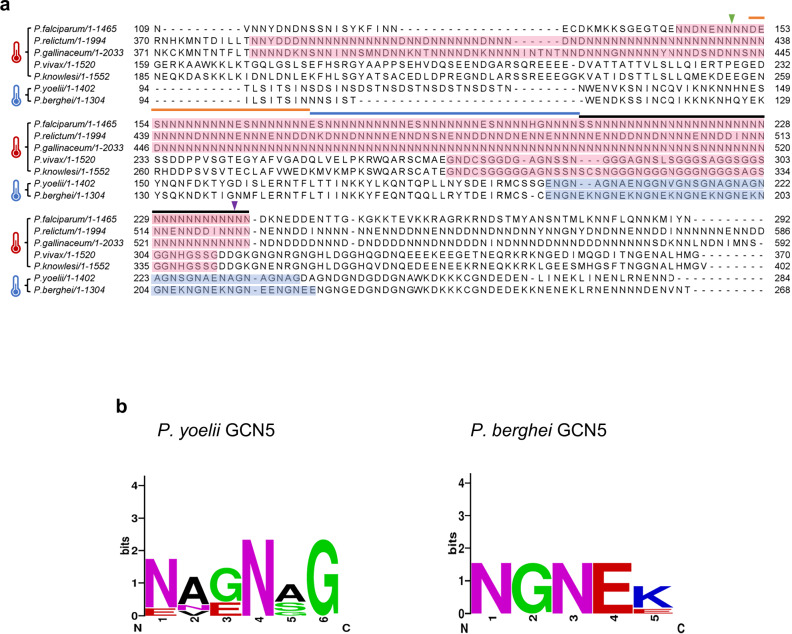
Low- and high-complexity repeats in the N-termini of plasmodial GCN5 homologs. **a** Low-complexity regions containing poly-N or GS-rich repeats in parasites naturally exposed to fever during their intraerythrocytic life cycle, highlighted in pink. Short repeats of higher complexity found in parasites that do not encounter fever during their intraerythrocytic life cycle, highlighted in blue. Thermometer cartoons in red and blue indicate whether hosts experience fever or not, respectively. The black line represents the sequence deleted in Del1 (amino acid position 206–240); the blue line represents additional sequence deleted in Del2 (amino acid position 173–240); the orange line depicts additional sequence deleted in Del3 lines (amino acid position 152–240). Therefore, the deleted region in Del3 encompasses the combined length of the three lines. The green and purple arrowheads before amino acid residues 150 and 238 represent the HiBiT insertion site in the Del3-HiBiT and LgBiT-HiBiT lines, respectively. This alignment was generated using Jalview, powered by JABAWS^78^. **b** Sequence logo of short repeats found in GCN5 homologs from *P. yoelii* and *P. berghei*. These images were generated using the web-based application WebLogo 3^79^.

GCN5 is a highly conserved protein across eukaryotes, with most of the homology restricted to its C-terminus, where the HAT domain and the BrD are located (Supplementary Fig. 1). Yeast and Tetrahymena GCN5 have short N-termini with less than 200 amino acids upstream of the HAT domain. In contrast, metazoans express two GCN5 isoforms from alternative splicing: a short version that is highly homologous to the yeast GCN5 and a long variant whose extended N-terminus displays a P300/CBP-associated factor (PCAF)-homology domain^21–24^. The PCAF-homology domain has been implicated in the recruitment of transcriptional activators, including two other HAT proteins, P300 and CBP, recognition of nucleosomal substrates, and it has been shown to possess E3 ubiquitin ligase activity^21,25^. The PfGCN5 (PF3D7_0823300) is produced as a single transcript and exhibits the longest N-terminal extension described to date. This extension contains no recognizable domains except for a 98-residue imperfect repeat containing 81 N (spanning from amino acid position 143 to 240) (Fig. 1a, Supplementary Fig. 2). The related apicomplexan parasite, *Toxoplasma gondii*, encodes two GCN5 homologs (TgGCN5-A/B) that are expressed as single transcripts and exhibit extended N-terminal extensions as well. However, these N-terminal extensions share little sequence similarity with PfGCN5^26,27^.

Sequence alignment of GCN5 homologs across *Plasmodium* species revealed that those naturally exposed to febrile temperatures, such as *P. falciparum*, *P. vivax*, *P. knowlesi*, *P. gallinaceum*, and *P. relictum*, possess N-terminal low complexity intrinsically disordered regions (LC-IDRs) consisting of poly-N or glycine-serine (GS)-rich repeats (Fig. 1a, Supplementary Fig. 2). In contrast, GCN5 homologs from *Plasmodium* species that do not experience fever, such as *P. yoelii* and *P. berghei*, display shorter and less conspicuous repetitive sequences rich in N, G, E, and A (Fig. 1a, b). Notably, avian malaria parasites (*P. gallinaceum* and *P. relictum*) survive their host’s body temperature, ranging between 38.5 and 43.8 °C, and rodent malaria parasites (*P. berghei* and *yoelii*) experience hypothermia instead of hyperthermia^28–30^. These observations led us to hypothesize that the extended LC-IDRs in the N-terminus of *Plasmodium* spp. naturally exposed to fever may confer a survival advantage in their hosts.

**Fig. 2:**
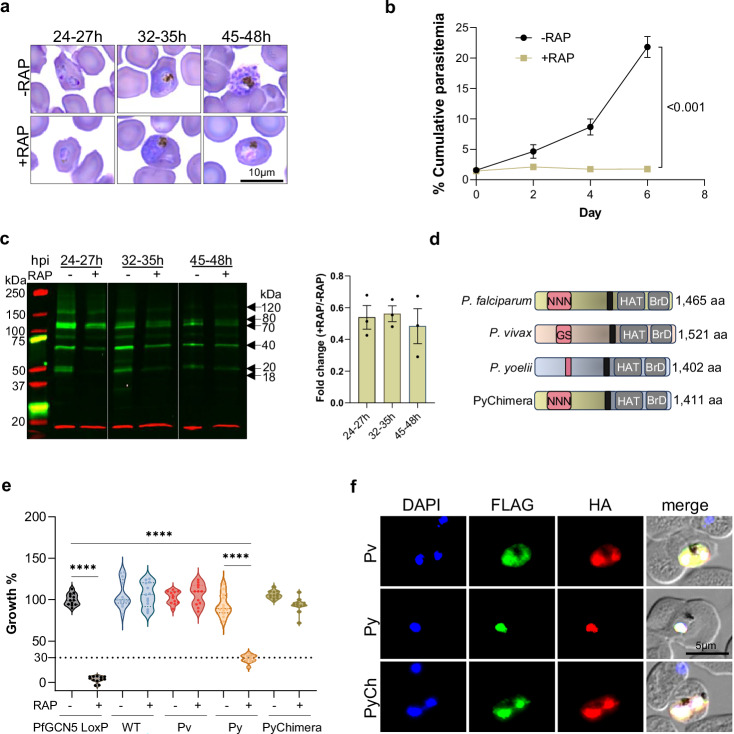
The N-terminus of PfGCN5 containing the poly-asparagine motif is required for parasite growth. **a** Blood smears from PfGCN5^LoxP^ parasites treated with or without rapamycin (RAP). Representative images from three independent experiments. **b** Growth of PfGCN5^LoxP^ parasites treated with or without RAP, measured by flow cytometry every 2 days over 6 days. Mean values and error bars representing the standard deviation (SD) from three independent experiments are shown. Data were analyzed statistically by a two-tailed Student’s *t*-test. **c** Representative western blot from three biological replicates illustrating the differences in protein expression of PfGCN5^LoxP^ parasites treated with or without RAP at 3 h post-infection (hpi). An anti-HA antibody was used to recognize the C-terminally processed forms of endogenous PfGCN5 (green), and an anti-histone H3 antibody was used as a loading control (red). Right bar graph: expression was quantified by densitometry after normalizing the HA signal to that of H3. Shown is the mean fold change between +RAP/-RAP ± SD of three biological replicates. **d** Schematic representation of plasmodial GCN5 homologs and chimeric construct. In gray are the histone acetyltransferase (HAT) domain and bromodomain (BrD), in black is the conserved motif used to delimit the PfGCN5 N-terminus in the chimera, in pink are the repeats, and in gradient colors are the unique sequences for each species. **e** Growth of rescue lines with or without RAP relative to the control (PfGCN5^LoxP^ parasites without RAP) after three intraerythrocytic cycles. The distribution of three or four independent experiments is shown. Dashed lines inside violins represent, from top to bottom, the third quartile, the median, and the first quartile. Data were analyzed statistically by one-way ANOVA with Tukey’s multiple comparison post hoc test. ****p = <0.0001 (multiplicity-adjusted *p*-value, 95% CI). **f** Immunofluorescence analysis of rescue lines without RAP, showing colocalization between the endogenous PfGCN5 protein identified with anti-HA antibodies (red) and the second copy protein identified with anti-FLAG antibodies (green). DAPI was used as a nuclear marker. Shown are representative images of three biological replicates. Pv: *P. vivax*, Py: *P. yoelii*, PyCh: PyChimera. Source data are provided as a Source Data file.

To test this hypothesis, we generated a rapamycin-inducible PfGCN5 conditional knockout line (PfGCN5^LoxP^) using the DiCre recombinase system^31^. Specifically, we integrated a *Saccharomyces cerevisiae*-recoded version of the *pfgcn5* coding sequence, C-terminally GFP-3HA tagged and flanked by LoxP sites, at the endogenous locus using CRISPR/Cas9 (Supplementary Fig. 3a). By employing an NF54 *attB* line expressing a dimerizable Cre recombinase, we could excise the entire gene upon rapamycin (RAP) addition (Supplementary Figs. 2, 3b)^32,33^. Similar to previous reports, before excision, we observed nuclear localization of PfGCN5 throughout the intraerythrocytic cycle and extensive processing of the full-length protein (190 kDa), resulting in multiple fragments (120, 80, 70, 40, 20, and 18 kDa) (Supplementary Fig. 3c, d)^16,34^. Of note, the protein is synthesized starting at the early ring stage and is almost completely processed by the end of the intraerythrocytic cycle. Following RAP addition (0–3 h post-invasion, early ring stage), knockout (KO) parasites stalled at the trophozoite stage and were unable to undergo schizogony, resulting in cell death (Fig. 2a, b). Time-course western blotting analysis showed that protein expression was reduced by approximately 50% in RAP-treated cultures at 24 h and remained stable for the remainder of the cycle, highlighting the stability of these fragments (Fig. 2c). We then attempted to rescue the PfGCN5 KO parasites with a series of C-terminally FLAG-tagged constructs expressed in trans from the *attB* locus under the control of the native *pfgcn5* promoter (Fig. 2d, Supplementary Fig. 4)^32^. Growth and development could be fully rescued by the introduction of a second copy of the WT *pfgcn5* gene (Fig. 2d, e). Interestingly, while *P. vivax* (Pv) GCN5 (PVX_089200) fully rescued the growth of PfGCN5 KO parasites, *P. yoelii* (Py) GCN5 (PY17X_0707600) supported only partial rescue, displaying ~30% growth over three replication cycles (Fig. 2d, e). To evaluate whether this growth phenotype was due to the N-terminus of PyGCN5, we made a chimeric protein containing the first 676 aa from the PfGCN5 N-terminus, including the poly-N repeat, and the C-terminus from PyGCN5. Given the widespread processing of PfGCN5, we determined the length of the PfGCN5 N-terminus based on the conserved motif IKNI/M/LR found at amino acid residue 677 (Supplementary Fig. 2). This chimeric protein was able to fully rescue the growth of PfGCN5 KO parasites (Fig. 2d, e). Finally, we observed that all GCN5 homologs and the PyChimera were localized to the nucleus, well expressed, and processed (Fig. 2f and Supplementary Fig. 4c). Taken together, these results suggest that the N-termini of PfGCN5 and PvGCN5, containing poly-N or GS-rich repeats, respectively, are important for *P. falciparum* parasite survival.

### The poly-N repeat in PfGCN5’s N-terminus is required for normal parasite growth

**Fig. 3:**
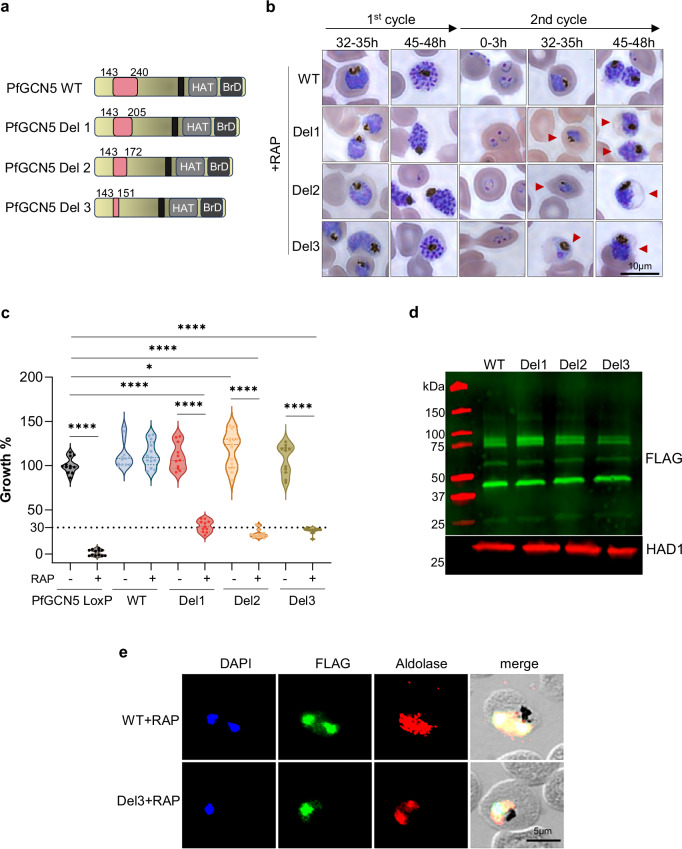
The poly-N domain in PfGCN5 is required for parasite growth. **a** Schematic representation of deletion constructs. The pink boxes illustrate the quantity of asparagine residues that remain: 48 N in Del1, 23 N in Del2, and 7 N in Del3 out of 81 N in the WT copy. Numbers above the pink boxes are the boundary residues of the repeat. PfGCN5 constructs were expressed under the control of the native promoter as second copies in the PfGCN5^LoxP^ conditional knockout line. **b** Giemsa-stained blood smears of deletion rescue lines treated with RAP across two life cycles. Red arrowheads point to aberrant cells. Representative images from three independent experiments with similar results. **c** Growth of WT and deletion rescue lines relative to the control (averaged growth of PfGCN5^LoxP^ parasites in the absence of RAP) after three intraerythrocytic cycles. The distribution of three independent experiments is shown. Dashed lines inside violins represent, from top to bottom, the third quartile, the median, and the first quartile. Data were analyzed statistically by one-way ANOVA with Tukey’s multiple comparison post hoc test. *p = 0.0106, ****p = <0.0001 (multiplicity-adjusted *p*-values, 95% CI). **d** Representative western blot from three biological replicates showing FLAG-tagged GCN5 protein levels in WT and deletion rescue lines (green). HAD1: sample processing control (red). **e** Immunofluorescence analysis showing nuclear localization for both WT and truncated PfGCN5 (Del3) proteins, identified with anti-FLAG antibodies. DAPI and aldolase were used as markers for the nucleus and cytoplasm, respectively. Shown is one biological replicate from three independent experiments. Source data are provided as a Source Data file.

To dissect whether the growth-related role of PfGCN5 N-terminus lies in its poly-N repeat, we engineered three C-terminally FLAG-tagged PfGCN5 rescue constructs, whose poly-N repeat was incrementally shortened from its C-terminal end (Fig. 3a, Supplementary Fig. 5). These deletions retained varying lengths of the 98-residue repeat containing 81 N found in the WT protein: deletion #1 (Del1) retains 48 N, deletion #2 (Del2) 23 N, and deletion #3 (Del3) 7 N. As before, these constructs were integrated at the *attB* site of the PfGCN5^LoxP^ line. Upon RAP treatment, unlike the death phenotype of PfGCN5 KO parasites, the deletion lines could progress through the first cycle without any notable defects, presenting abnormal trophozoite and schizont morphologies only in the second cycle (Fig. 3b). Similar to the PyGCN5 rescue line, all three deletion lines partially rescued the growth of PfGCN5 KO parasites, exhibiting about 30% growth relative to the WT line over three replication cycles (Fig. 3c). Western blot analysis confirmed that all deletion constructs were expressed at comparable levels to the WT protein and underwent normal proteolytic processing (Fig. 3d). Based on the consistent phenotype, proper protein level expression, and processing across the deletion lines, we selected one line (Del3) for the subsequent analysis. As in the WT, the Del3 rescue line showed nuclear localization of the second copy of PfGCN5 (Fig. 3e). Altogether, these data indicate that the poly-N repeat in PfGCN5 is required for normal parasite growth.

### The poly-N repeat in PfGCN5 facilitates cell recovery after stress

**Fig. 4:**
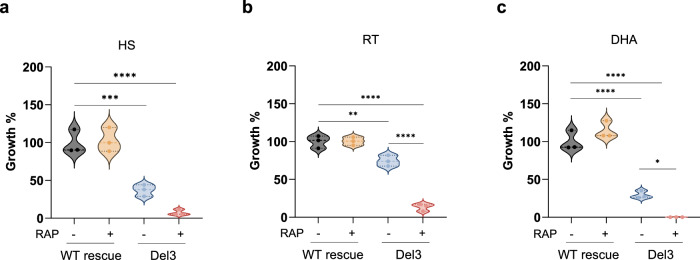
The PfGCN5 deletion line is susceptible to stress. Synchronized 0–3 h post-invasion PfGCN5^LoxP^ parasites expressing a PfGCN5 WT or deletion (Del3) second copy were treated with or without RAP, and 24 h later were exposed to **a** heat shock (HS, 41 °C, 6 h), **b** room temperature (RT, 25 °C, 12 h), or **c** dihydroartemisinin (DHA, 100 nM, 90 min) before returning to standard conditions. The growth percentage was calculated as the final parasitemia after three intraerythrocytic cycles, normalized to that of the WT rescue line grown in the absence of RAP. The distribution of three independent experiments is shown. Dashed lines inside violins represent, from top to bottom, the third quartile, the median, and the first quartile. Data were analyzed statistically by one-way ANOVA with Tukey’s multiple comparison post hoc test. *p = 0.0161, **p = 0.0061, ***p = 0.0010, ****p = <0.0001 (multiplicity-adjusted *p*-values, 95% CI). Source data are provided as a Source Data file.

The observation that poly-N or GS-rich repeats are found in GCN5 homologs from *Plasmodium* spp. that are naturally subjected to fever but absent in those that do not encounter such conditions prompted us to investigate the potential link between these LC-IDRs and stress. Numerous reports in *P. falciparum* and other systems have emphasized GCN5’s ability to act as a master regulator of stress by deploying transcriptional cascades that enable stress management^15,16,35–37^. Specifically, HS, low glucose, and dihydroartemisinin (DHA) have been shown to induce higher PfGCN5 expression^15,16,38^. Additionally, restricting PfGCN5 overexpression heightens the parasite’s susceptibility to stress^15,16^. With this in mind, we subjected our WT and Del3 rescue lines to HS (41 °C for 6 h), room temperature (RT) (~25 °C for 12 h), and DHA (100 nM for 90 min) starting 24 h after a 3 h RAP pulse at the 0–3 h ring stage^39–41^. We then returned the cultures to standard conditions (37 °C, drug washed out) for 6 days. Exposure to HS and RT exacerbated the growth defect in RAP-treated Del3 parasites in comparison to the WT line and untreated Del3 parasites (Fig. 4a, b). Exposure to DHA rendered RAP-treated Del3 parasites unable to recover (Fig. 4c). Intriguingly, Del3 parasites subjected to HS and DHA in the absence of RAP also exhibited poor recovery. This dominant-negative effect could result from the increased expression of PfGCN5 during stress, leading to the displacement of the WT protein by the truncated version in SAGA-like complexes. These results support the hypothesis that LC-IDRs in the N-terminus of PfGCN5 contribute to the parasite’s adaptive transcriptional regulation.

### PfGCN5 histone acetyltransferase activity is supported by its poly-N repeat

**Fig. 5:**
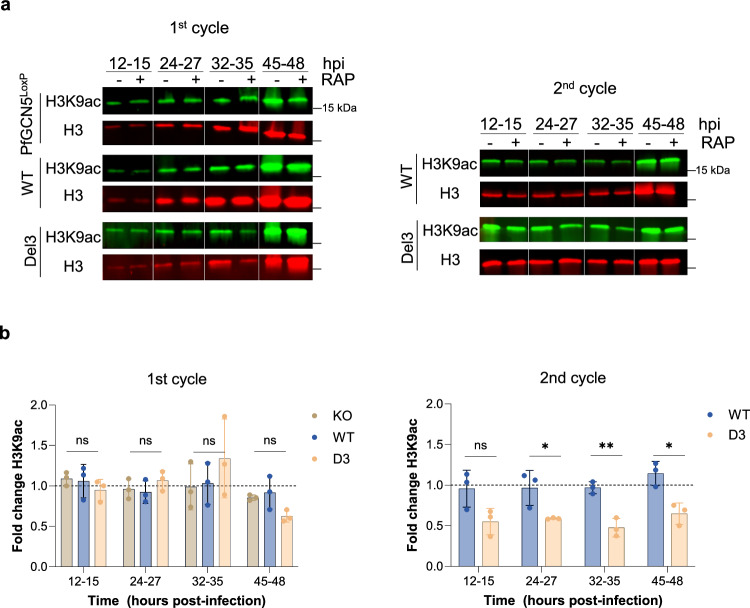
Deletion of the Poly-asparagine repeat affects PfGCN5-mediated acetylation of H3K9ac. **a** Unmodified H3 and H3K9ac levels were identified with anti-histone H3 and anti-H3K9ac specific antibodies in protein lysates from parasites treated with or without RAP. Shown are representative western blots from three biological replicates processed in parallel. Lines on the right side of each blot indicate the 15 kDa molecular weight mark. **b** Quantification of the fold change between normalized H3K9ac levels to unmodified H3 from RAP-treated and non-treated parasites from three biological replicates. Data are presented as mean values ± SD. Data from the 1st cycle were analyzed statistically by one-way ANOVA with Tukey’s multiple comparison post hoc test. Data from the 2nd cycle were analyzed statistically by a two-tailed Student’s *t*-test (PfGCN5 KO parasites were dead by the second cycle and thus excluded from these analyses). *p = 0.039, **p = 0.0032 (multiplicity-adjusted *p*-values). Source data are provided as a Source Data file.

**Fig. 6:**
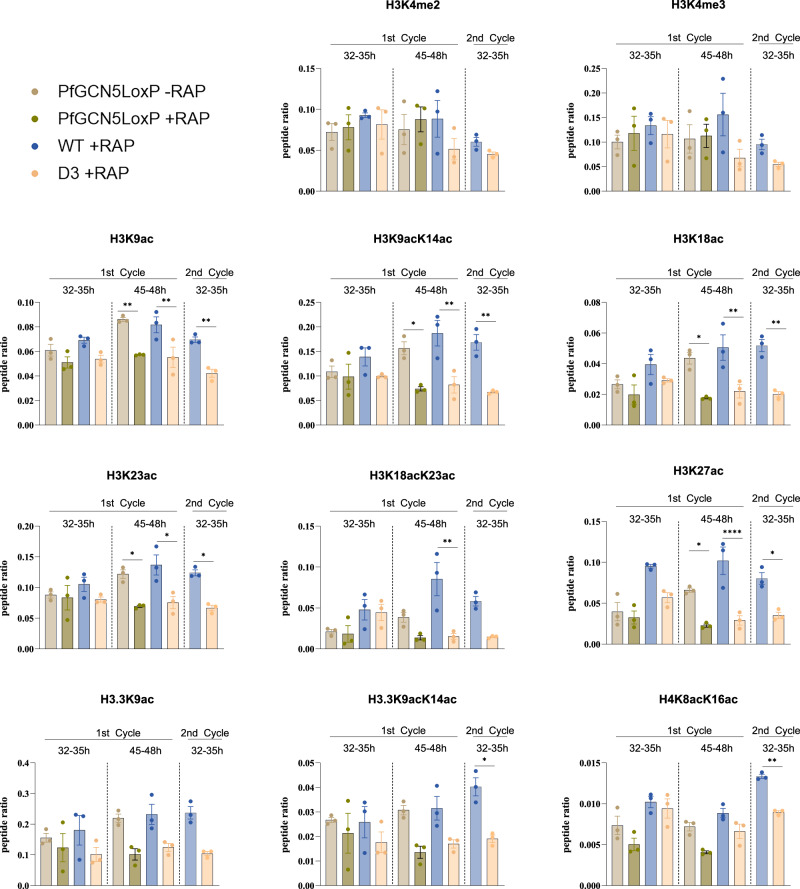
Histone acetylation modifications identified by mass spectrometry. Acid-extracted core histones were subjected to propionylation and trypsin digestion before LC-MS/MS analysis. The total abundance of each modified peptide was calculated based on the sum of all unmodified and modified peptide forms. The ratio of each peptide was determined as its abundance relative to the total abundance. Mean values ± the standard error of the mean (SEM) from three biological replicates are shown. Data were analyzed statistically by one-way ANOVA with Fisher’s multiple comparisons post hoc test. *p = 0.0152, **p = 0.0022, ***p = 0.0005, ****p = <0.0001 (multiplicity-adjusted *p*-values, 95% CI). Source data are provided as a Source Data file.

PfGCN5 retained HAT activity when its C-terminal region, comprising the HAT and BrD or the HAT domain alone, was used to acetylate free histone H3 at lysine residues 9 and 14 (H3K9ac and H3K14ac) from calf thymus^19^. Subsequent studies confirmed this activity in parasites^17^. Although *P. falciparum* encodes four histone variants (H2A.Z, H2B.Z, H3.3, and CenH3) besides the canonical core histones, PfGCN5’s role in these variants is unknown, except for H2B.Z, whose acetylation levels were lower in a PfGCN5 BrD deletion line^18,42^. To investigate if the poly-N repeat in PfGCN5 could play a role in histone acetylation, we examined H3K9ac levels in synchronized parasites treated with or without rapamycin at 0–3 h post-invasion. Samples were harvested at 12–15 h, 24–27 h, 32–35 h, and 45–48 h post-invasion during the first intraerythrocytic cycle post-RAP addition at 0–3 h in the same cycle. While we observed slight decreases in the level of H3K9ac at 45–48 h in the PfGCN5^LoxP^ and Del3 lines treated with RAP, there were no significant differences across lines at any of the evaluated time points (Fig. 5a, b, left side). Encouraged by this trend and by the observation that RAP-treated deletion lines exhibited a fitness cost only during the second cycle, we extended these studies to the second intraerythrocytic cycle post-rapamycin addition. In agreement with previous studies, we found significant changes in H3K9ac at all time points during the second cycle in RAP-treated Del3 parasites (Fig. 5a, b, right side)^17,19^. Note that PfGCN5 KO parasites die in the first cycle, so acetylation levels in the second cycle cannot be assessed. In light of the limitations of western blot analysis for quantitation and the limited availability of anti-histone antibodies for examining other histone modifications, we opted for a quantitative, high-resolution mass spectrometry approach. To achieve this, we purified *P. falciparum* core histones using an acid extraction method from synchronized cultures at 32–35 h and 45–48 h of the first intraerythrocytic cycle, and at 32–35 h of the second intraerythrocytic cycle post-RAP induction. Interestingly, we found significant differences for H3K9ac, H3K9acK14ac, H3K18ac, H3K23ac, H3K18acK23ac, H3K27ac, H3.3K9acK14ac, and H4K8acK16ac at 45–48 h of the first intraerythrocytic cycle and/or at 32–35 h of the second intraerythrocytic cycle (Fig. 6). We did not observe any differences in H3K9ac levels at 32–25 h of the first intraerythrocytic cycle, consistent with our western blot results. The non-significant trend toward decreased H3K9ac in the western blot studies became significant differences at 45–48 h of the first intraerythrocytic cycle for the MS analysis, underscoring the technique’s sensitivity. Notably, H2B.Z was not detected, and di- and tri-methylation of H3K4 did not show significant differences across lines, in contrast to the western blot findings of a previous paper^17^. Overall, these findings confirm and broaden our understanding of the extensive role of PfGCN5 in chromatin remodeling and gene expression. More critically, they suggest that the poly-N repeat contributes to the HAT activity of PfGCN5.

### The PfGCN5 deletion protein is fully enzymatically active

**Fig. 7:**
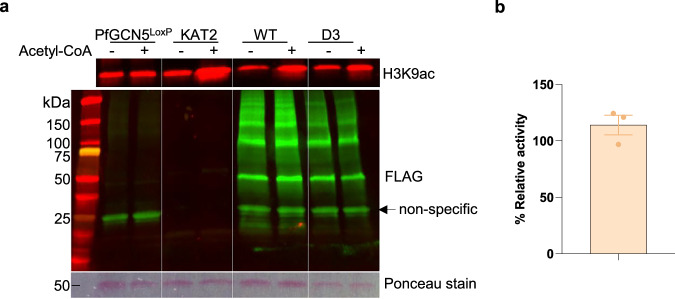
PfGCN5 WT and Del3 proteins isolated from parasites demonstrated equal histone acetyltransferase activity. **a** Purified proteins were incubated with 10 µg of calf thymus histones and 60 µM of acetyl-CoA or water for 1 h at 30 °C. Reactions were analyzed by western blotting using anti-H3K9ac and anti-FLAG antibodies to quantitate acetylation and PfGCN5 proteins, respectively. The H3K9ac signal in the absence of acetyl-CoA was subtracted from their corresponding comparator in the presence of acetyl-CoA. Net H3K9ac signal was then normalized to the corresponding amount of pulled-down protein (FLAG blot). PfGCN5^LoxP^ parasites were used as a negative control and the recombinant human GCN5 (KAT2) protein as a positive control. Ponceau-stained bands were used as loading controls for the FLAG blot (same membrane). **b** Quantitation of three biological replicates where the activity of the Del3 protein was calculated as a percentage of the WT protein activity. Mean values ± SEM are shown. A two-tailed Student’s *t*-test described non-significant differences (*p* = 0.25). Source data are provided as a Source Data file.

We isolated the FLAG-tagged second copy of PfGCN5 from WT and Del3 rescue lines and tested their activity in a histone acetyltransferase assay in vitro. Both the WT and Del3 proteins are equally active in the presence of acetyl-CoA (Fig. 7). This suggests that other factors are responsible for the diminished acetylation activity found for the deletion version of PfGCN5 in intact parasites.

### PfGCN5 is processed in the nucleus

**Fig. 8:**
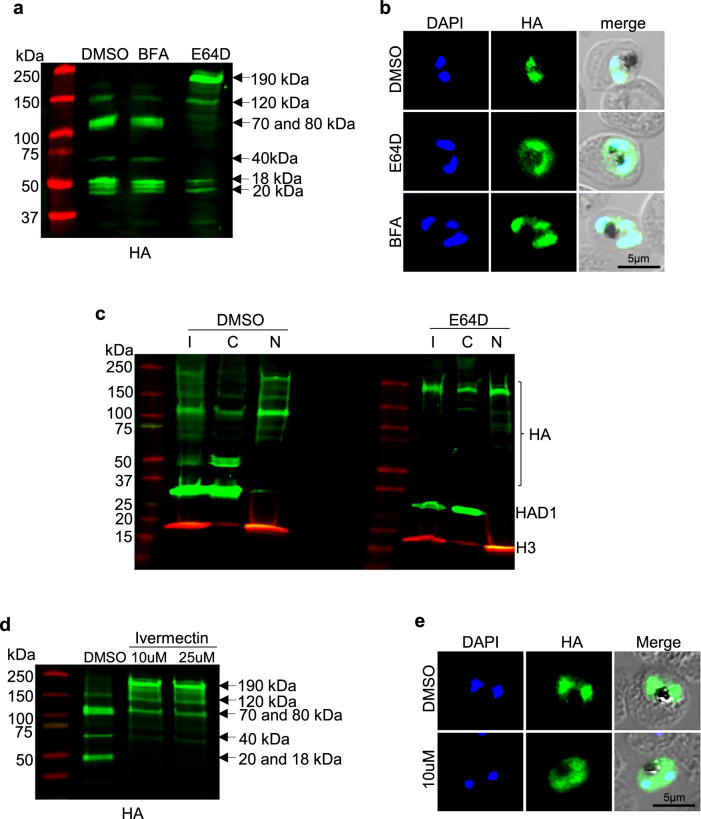
PfGCN5 does not require processing prior to nuclear localization. **a** Representative anti-HA immunoblot from three biological replicates showing processing of the endogenous PfGCN5 protein in the PfGCN5^LoxP^ line in the absence of rapamycin. Arrows represent the molecular mass of the C-terminal fragments after subtracting the mass of the GFP tag. **b** Immunofluorescence analysis showing cells from the PfGCN5^LoxP^ line in the absence of rapamycin, treated with E64D (10 μM), brefeldin A (BFA, 0.5 μg/mL), or DMSO. Three biological replicates were performed. **c** Cells treated with DMSO or E64D for 3 h were fractionated. Anti-HAD1 and anti-histone H3-specific antibodies were used as markers of the cytosolic “C” and nuclear “N” fractions, respectively. “I” = input. Shown is a representative western blot from three biological replicates. **d** Representative western blot from two biological replicates showing enrichment of the full-length protein upon treatment of parasites with 10 μM or 25 μM ivermectin in comparison to the DMSO control. Arrows represent the molecular mass of the C-terminal fragments identified with an anti-HA antibody after subtracting the mass of GFP. **e** Localization of PfGCN5 in cells treated with ivermectin or DMSO. DAPI and anti-HA antibodies were used to localize the nucleus and endogenous PfGCN5 protein, respectively. Representative images from three independent experiments with similar results.

Recent studies have proposed that PfGCN5 is trafficked via the ER-Golgi to the digestive vacuole, where it is processed by the cysteine protease, falcipain 3, before translocating to the nucleus^34,43^. We, however, did not find evidence of this. We treated asynchronous PfGCN5^LoxP^ parasites with the cysteine protease inhibitor, E64D (10 μM), the secretory pathway inhibitor Brefeldin A (5 µg/mL), or DMSO for 3 h^44,45^. While parasites treated with E64D displayed an enrichment of the full-length protein as previously reported, PfGCN5 nuclear localization remained unchanged (Fig. 8a, b)^34^. This outcome suggests that the full-length protein is processed in the nucleus. Furthermore, we observed that PfGCN5 processing and trafficking are insensitive to Brefeldin A treatment, indicating an ER-Golgi-independent trafficking pathway. Fractionation analysis of DMSO or E64D-treated cells confirmed that the full-length protein does not require processing before entering the nucleus (Fig. 8c).

The importin α/β machinery mediates a major nuclear transport pathway. Given that TgGCN5A enters the nucleus via direct interaction with the adaptor molecule importin-α, we sought to test whether PfGCN5 uses this pathway^27^. We treated asynchronous PfGCN5^LoxP^ parasites with 10 μM or 25 μM ivermectin for 3 h. Treatment of *P. falciparum* parasites with ivermectin has been shown to block this pathway^46^. We found that ivermectin-treated parasites not only exhibit stabilization of the full-length protein but also redistribution of the PfGCN5 signal to the cytoplasm, in stark contrast to DMSO-treated parasites (Fig. 8d, e).

### The poly-N repeat confers stability to the N-terminal polypeptide post-cleavage and interacts with the C-terminal catalytic domain

**Fig. 9:**
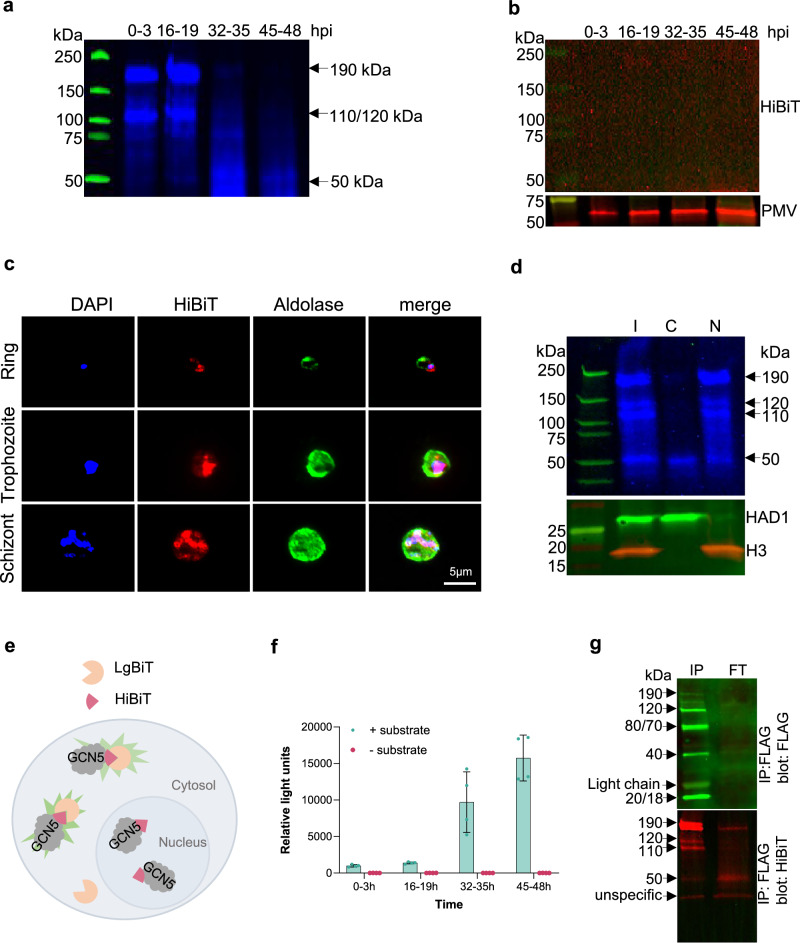
Intracellular dynamics of the N-terminal polypeptide of PfGCN5. Time-course luminescence blotting of protein lysates from **a** LgBiT-HiBiT and **b** Del3-HiBiT lines. Representative blots from three independent biological replicates. The PMV signal was used as a sample processing control. **c** Localization of PfGCN5 HiBiT-tagged fragments by IFA using an anti-HiBiT antibody. DAPI and aldolase were utilized to stain the nucleus and cytoplasm, respectively. Shown is one biological replicate from three. **d** Luminescence blotting of fractionated LgBiT-HiBiT parasites at 20–23 hpi. Anti-HAD1 and anti-histone H3-specific antibodies were used as markers of the cytosolic “C” and nuclear “N” fractions, respectively. The bottom blot was run separately and was used as an assessment of the fractionation. “I” = input. Shown is one biological replicate from three. **e** Illustration of cytosolic recognition of HiBiT-tagged fragments by the LgBiT subunit. The nucleus-impermeable LgBiT protein cannot probe nuclear fragments tagged with HiBiT, only cytosolic fragments. **f** Live-cell detection of cytosolic HiBiT-tagged fragments throughout the intraerythrocytic life cycle. Mean values ± SD from four independent experiments are shown. **g** Immunoprecipitation of C-terminal fragments was performed using mouse anti-FLAG antibodies conjugated to magnetic beads. After detection of the FLAG signal, the same membrane was used in luminescence-based detection of HiBiT. Representative blot from three independent replicates. IP immunoprecipitate, FT flow-through. Labels to the right indicate the tags used for western blotting. Source data are provided as a Source Data file.

We wanted to tag the N-terminus of PfGCN5 to track its kinetics. After several unsuccessful attempts, we adopted the NanoLuc® Binary Technology, NanoBiT (Promega), for higher-resolution monitoring of the poly-N repeat. For this, we first engineered a stable parasite line expressing the larger NanoLuc subunit, LgBiT, using the *piggyBac* transposon-mediated genomic integration^47^. Whole-genome sequencing of two clones and the parental line revealed that the *lgbit* gene was inserted at the start of the Hsp70/Hsp90 organizing protein (PF3D7_1434300) at position 1,372,910 bp of chromosome 14 (Supplementary Fig. 6a). Growth assays showed no significant differences between a WT and the LgBiT-expressing line (Supplementary Fig. 6d). Expression and localization of LgBiT were verified by western blot and IFA, respectively, revealing an 18 kDa cytosolic protein that is consistent with findings in other systems (Supplementary Fig. 6b, c)^48^. We then used this LgBiT line to integrate a C-terminally FLAG-tagged second copy of PfGCN5 containing the 11-amino acid smaller NanoLuc subunit, HiBiT, in the poly N-repeat (Fig. 1a) at the *attB* site as before, resulting in the LgBiT-HiBiT line. To regulate expression, this second copy of PfGCN5 was placed under the control of its endogenous promoter. Insertion of HiBiT did not impair parasite growth in comparison to the WT line (Supplementary Fig. 6d). Similarly, we attempted to introduce the truncated version of PfGCN5, Del3, tagged with HiBiT (Fig. 1a) in the LgBiT line, but were unsuccessful. Therefore, we introduced this second copy into the PfGCN5^LoxP^ line, resulting in the Del3-HiBiT line. This line also exhibited normal growth (Supplementary Fig. 6d). Luminescence-based detection of the WT HiBiT-tagged protein by western blot revealed the presence of the 190 kDa full-length protein, and processed forms of 120, 110, and 50 kDa that were sequentially produced through the parasite intraerythrocytic cycle (Fig. 9a). Notably, all these fragments were also detected by another group using polyclonal antibodies that recognize the N-terminal region of PfGCN5, encompassing amino acid residues 9–25^15^. In contrast, in the Del3-HiBiT line, HiBiT western blotting failed to recognize bands, indicating that the N-terminal polypeptide is rapidly degraded (Fig. 9b). IFAs showed that the poly-N repeat containing fragments in the LgBiT-HiBiT line exhibit a higher concentration in the nucleus with a weak cytoplasmic signal in trophozoite and schizont stages (Fig. 9c). To investigate the dynamics of the PfGCN5 N-terminus in the cell, we performed cellular fractionation studies using the LgBiT-HiBiT line. Interestingly, the full-length, the 120 kDa, and the 110 kDa forms remain in the nucleus, while the 50 kDa fragment containing the poly-N repeat is exported to the cytosol (Fig. 9d). To further explore the live-cell kinetics of the poly-N-repeat containing fragments, we took advantage of the high affinity between the LgBiT and HiBiT subunits and employed a cell-permeable substrate (furimazine) to measure the real-time binding activity. We first confirmed that the LgBiT protein is nucleus impermeable even in the presence of the PfGCN5-HiBiT protein by performing cellular fractionation (Supplementary Fig. 6e). Since LgBiT was found to be exclusively cytosolic, it acts as a cytosolic sensor for HiBiT-tagged poly-N repeat-containing fragments (Fig. 9e). Consistent with our earlier observations in Fig. 9c, d, cytosolic levels of HiBiT-tagged fragments rise dramatically midway through the cycle, after the 50 kDa fragment is generated (Fig. 9f).

Given that the deletion of the poly-N repeat resulted in the destabilization of the N-terminal polypeptide post-cleavage and a reduction in histone acetylation levels, a function catalyzed by the C-terminal HAT domain, we wondered if the N-terminal polypeptide containing the poly-N repeat could associate with the C-terminal fragments. To address this question, we pulled down the C-terminal fragments by the FLAG tag and blotted for HiBiT luminescence (Fig. 9g). C-terminal FLAG-tagged forms could co-precipitate all HiBiT-tagged fragments, suggesting interaction. Notably, the 50 kDa HiBiT-tagged piece, previously shown to be exported to the cytosol, was more abundant in the flow-through than in the IP fraction, leaving the 120 kDa and 110 kDa fragments as the potential interactors of the catalytic domain. Additionally, we asked whether N-terminal peptides could be identified in C-terminal pull-downs by mass spectrometry and if shortening the length of the poly-N repeat in PfGCN5 disturbs complex formation or other interactions. To do this, we performed co-immunoprecipitation analysis using anti-FLAG magnetic beads across the WT and deletion rescue lines in the absence of rapamycin. LC-MS/MS analysis identified N-terminal peptides when pulling down from the WT rescue line but not from the deletion rescue lines, confirming the association between N- and C-terminal fragments in the WT line (Supplementary Fig. 7). While five out of nine PfGCN5 complex members were pulled down with both the WT and Del3 rescue lines, the stoichiometry of one of its members was dysregulated in the deletion lines (Supplementary Table 1)^17^. Specifically, the Nucleosome Assembly Protein (NAPS; PF3D7_0919000) had a three-fold increase in deletion lines. Furthermore, the adaptor protein 14-3-3I (PF3D7_0818200) and a PHD-domain-containing protein (PF3D7_0310200), previously found significantly enriched in PfGCN5 pull-downs, were several-fold enriched in the deletion lines^17^. These findings indicate that the N-terminal domain containing the poly-N repeat interacts with the C-terminal catalytic domain post-cleavage.

## Discussion

In this study, we report a functional role for a poly-N repeat in a *P. falciparum* protein. With approximately 1300 proteins containing poly-N repeats, this is the most prevalent characteristic of the *P. falciparum* proteome that has remained elusive in understanding the biology of this deadly parasite. While the function of such repeats may vary across individual proteins, our findings suggest an important regulatory function in this instance, which opens a new area of research in *Plasmodium* biology.

We highlighted the existence of extended LC-IDRs in the N-terminus of GCN5 homologs from *Plasmodium* species that are naturally exposed to fever, in stark contrast to those that do not encounter this stress. Remarkably, PvGCN5, which carries a GS-rich repeat, rescued the growth of PfGCN5 KO parasites, and deletion of the poly-N repeat from PfGCN5 phenocopied the growth profile of the PyGCN5 (short higher-complexity repeat) rescue line. This suggests that the functional contribution of the N-terminal LC-IDR does not depend strictly on N residues but rather on the biophysical properties shared by polar and uncharged amino acid repeats. Thus, *Plasmodium* species may utilize synonymous “amino acid grammar” to achieve similar biological outcomes within their shared host. Mounting data in other systems has demonstrated the capacity for polar tracts rich in N, Q, G, and S to phase separate through dynamic multivalent interactions^49–51^. This phenomenon aids cellular organization, transcriptional regulation, genome maintenance, and complex formation, among others^52,53^. Whether LC-IDRs composed of poly-N repeats in *P. falciparum* phase separate remains to be solved. Intriguingly, 7 of 9 proteins forming the PfGCN5 SAGA-like complex also contain prominent poly-N repeats^17^. Some of these may enhance interaction among complex members, provide better anchoring to histones, or allow transient interactions during stress conditions. Indeed, we found that the Del3 protein failed to maintain the stoichiometry levels of one complex member (NAPS) in comparison to the WT protein. Furthermore, we observed that 14-3-3I and a PHD-containing protein were several-fold enriched in the Del3 line. 14-3-3I is a scaffold protein that binds to phosphorylated serine/threonine residues, modulating the function, localization, and stability of its binding partners, as well as stabilizing protein complexes by facilitating protein-protein interactions^54,55^. Given that PfGCN5 is extensively phosphorylated, we speculate that 14-3-3I may maintain the complex in tight engagement by binding to PfGCN5 and members of the SAGA-like complex simultaneously^56^. Perhaps increased 14-3-3 in Del3 parasites may be due to the exposure of a greater number of phosphorylated sites upon removal of the poly-N repeat.

It has been proposed that poly-N repeats in *P. falciparum* can behave as “tRNA sponges” by slowing down ribosomal translation, giving the expected limited availability of asparaginylated tRNAs^57^. However, we demonstrated that the PfGCN5 Del3 truncated protein is expressed at the same level as the WT second copy, suggesting that translation efficiency did not depend on the amount of N residues. Extensive truncation studies in the N-terminal extension of TgGCN5-A/B identified divergent nuclear localization signals embedded in intrinsically disordered regions^58^. In PfGCN5, we demonstrated that, like the WT, the Del3 mutant PfGCN5 localizes to the nucleus, indicating that the poly-N repeat in PfGCN5 does not mediate nuclear trafficking. Together, we established that reducing the length of the poly-N repeat in PfGCN5 does not affect expression levels, processing, or localization.

Substantial data support the essential role of GCN5 during stress by remodeling chromatin structure through histone acetylation and by binding to a subset of genes directly implicated in stress tolerance, in both *P. falciparum* and other organisms^15,16,59,60^. We showed that the Del3 line is highly susceptible to heat, cold, and drug stress, suggesting that the poly-N repeat aids PfGCN5 activity during stress, but further investigation is needed to uncover the molecular mechanism behind this.

Cellular fractionation studies examining the N-terminus, in conjunction with observations from E64D and Ivermectin treatments, demonstrated that the full-length PfGCN5 is exported to the nucleus, likely via the importin α/β transport machinery, where a cysteine protease subsequently processes it. Luminescence-based western blotting coupled with live-cell kinetics using a split nano-luciferase system allowed us to conclude that once in the nucleus, the N-terminus containing the poly-N repeat is cleaved into 120 kDa and 110 kDa processed forms. Later, midway through the intraerythrocytic cycle, a 50 kDa fragment is produced and exported back to the cytosol. Given that PfGCN5 has cytosolic non-histone substrates, it is possible that such bidirectional trafficking of the poly-N repeat contributes to this^38,61^. Inspection of N- and C-terminal blots indicates that PfGCN5 is alternatively cleaved, producing a 120 kDa fragment from the N- or C-terminus.

Only H3K9ac, H3K14ac, H3K4me3, and H2B.Zac had been described as modifications catalyzed by PfGCN5^17,18^. Here, we extended the post-translational landscape mediated by PfGCN5 to H3K9acK14ac, H3K18ac, H3K23ac, H3K18acK23ac, H3K27ac, H3.3K9ac, H3.3K9acK14ac, and H4K8acK16ac. Although we did not find significant changes for H3.3K9ac, a conserved trend was observed between PfGCN5^LoxP^ parasites treated with or without rapamycin and the WT and Del3 complemented lines. We did not find evidence that PfGCN5 promotes methylation of H3. Occupancy of H3K9ac at putative transcriptional initiation sites correlates with that of PfGCN5, and results in stage-specific gene expression in the parasite^20^. Although the biological role of H3K9acK14ac is less understood, the methyltransferase protein, PfSET2, preferentially modifies nucleosomes bearing this co-occurring mark, suggesting that PfGCN5 could indirectly recruit methyltransferase proteins^62^. In mouse embryonic stem cells, H3K9acK14ac occurs with high prevalence along with H3K4me3 and H3K27ac to maintain genes silenced but poised for rapid activation^63^. Acetylation of H3K18 and H3K27 is found at transcriptionally active sites and is associated with the transcription factor AP2-I and the bromodomain protein PfBDP1 during schizont stages^18^. Although a middle-down proteomics approach identified H3K18acK23ac exclusively in mature gametocytes, our bottom-up proteomics approach detected this co-occurring mark in trophozoite and schizont stages, perhaps due to its higher sensitivity and the proximity of the residues^64^. Notably, in gametocytes, H3K18ac and H3K23ac were associated with AP2-G and NAPS, a member of the SAGA-like complex, suggesting an intimate relationship between GCN5 and AP2 transcription factors to enable stage-specific changes. The histone variant H3.3 is predominantly found in GC-rich euchromatic coding and subtelomeric repetitive regions, indicating a wide range of occupancy for PfGCN5^65^. Consistent with our results, similar proteomic methods have identified H3.3K9ac and H3.3K9acK14ac in trophozoites^64^. While we found a decrease of H4K8acK16ac in KO and Del3 parasites, acetylation of H4K8 and H4K16 is catalyzed by PfMYST and is dynamically found in both euchromatin and heterochromatin regions during the intraerythrocytic life cycle^66,67^. Although in vitro assays have demonstrated that PfMYST and PfGCN5 preferentially acetylate H4 or H3, respectively, recombinant PfGCN5 acetylates H4 to a lesser extent^66^. Moreover, of the four H4 acetylation marks tested elsewhere (K5, 8, 12, and 16), PfMYST displayed low acetylation of H4K16 by several orders of magnitude. It is therefore possible that PfGCN5 facilitates the dual H4K8acK16ac mark by acetylating H4K16ac either directly or indirectly. One known effect of H4K8ac in heterochromatin regions is to promote expression of the antigenic *var* gene family^66,67^. These findings indicate that PfGCN5 plays a crucial role in writing the “chromatin code.” Histone acetylation changes in the Del3 line underpin the importance of the poly-N repeat during its stay in the nucleus.

Despite finding decreased histone acetylation in KO and Del3 parasites by high-resolution mass spectrometry and western blotting, the PfGCN5 Del3 protein displayed similar activity to that of the WT protein in a histone acetyltransferase in vitro assay. This indicates that other factors governing chromosomal architecture or nucleosomal activity, such as recognition, loading, and/or processivity, could impact the activity of the poly-N deletion protein in vivo. The enriched interaction of NAPS, a protein promoting chromosomal fluidity, with the Del3 protein hints at decreased accessibility or dynamism^68^. This work determined that the poly-N repeat stabilizes the N-terminal polypeptide following cleavage, which then associates with the C-terminal catalytic domain to support histone acetylation. Specifically, we identified that the 120 and/or 110 kDa N-terminal fragments, which only reside in the nucleus and are apparent during the first half of the cycle, are associated with the C-terminal catalytic domain. LC-IDRs can modulate stability by promoting conformational changes, protein interactions, or mediating subcellular localization^69,70^. Deletion of a non-poly-N-repeat LCR from a *P. falciparum* exonuclease limits substrate specificity^71^. Besides conferring stability and given its flexible nature, poly-N repeats could fine-tune transient interactions with complex members or substrates. While the molecular mechanism of the poly-N repeat contribution to histone acetylation remains to be identified, this study suggests that some poly-N repeats are evolutionarily favorable for the parasite.

## Methods

### Reagents

All primers were obtained from Integrated DNA Technologies. Recoded gene blocks (*pfgcn5*, *pvgcn5*, and *pygcn5*) were procured from Genewiz. Restriction enzymes and Gibson Assembly® Master Mix were purchased from New England Biolabs. In-fusion master mix was purchased from Takara. For site-directed mutagenesis, we used the Quick-Change Lightning kit from Agilent. The rabbit anti-HA antibody and the mouse anti-FLAG magnetic beads were obtained from Millipore Sigma. The rat anti-FLAG antibody was obtained from Biolegend. Rabbit anti-Plasmodium aldolase was from Abcam. The mouse anti-PMV and rabbit anti-HAD1 antibodies were as previously described^44^. The mouse anti-H3 and H3K9ac were obtained from Epigentek and Active Motif, respectively. Mouse anti-LgBiT and anti-HiBiT antibodies were obtained from Promega. Rapamycin, Brefeldin A, E64D, Dihydroartemisinin, Saponin, Sorbitol, WR99210, and the stain for thin smears (Hemacolor®) were purchased from Millipore Sigma. Histones from calf thymus (H9250) and Acetyl coenzyme A trisodium salt (A2056) were obtained from Millipore Sigma. Recombinant human KAT2A/GCN5 protein (NBP1-72426) was purchased from Novus Biologicals.

### Generation of plasmids

A *S. cerevisiae*-recoded version of *pfgcn5* was inserted at the AsiSI restriction site of the pSN054 plasmid, previously described by Polino et al.^72^. The GFP sequence amplified with primers KR24 and KR25 was inserted at the AsiSI restriction site in the pSN054. The AsiSI restriction site was restored upon cloning, allowing for a *S. cerevisiae*-recoded version of pfgcn5 amplified with primers KR137 and KR138 to be in frame with GFP and 3xHA, upon Gibson assembly. The 490 bp immediately upstream of the pfgcn5 start codon amplified with primer pair KR135 and KR136, and 792 bp downstream of the stop codon amplified with KR47 and KR21, were used as the left (LHR) and right homologous (RHR) region, respectively. The column-purified LHR and RHR were fused to restriction sites FseI and I-SceI, respectively, using Gibson assembly. This resulted in the pSN054_PfGCN5-GFP-3HA-LoxP plasmid that was used to modify the pfgcn5 locus. For generating rescue constructs, we used the pEOE-2X-attP-3xFLAG described elsewhere^45^. An upstream region, extending 987 bp from the pfgcn5 start codon, was amplified with primer pair KR104 and KR105 and utilized as a promoter. GCN5 variants amplified with the indicated primers in the Supplementary Data 2 file were introduced at the AvrII restriction site. All amplicons were introduced using In-Fusion cloning. To make the deletion constructs and HiBiT insertion, site-directed mutagenesis was employed using primers KR333, KR334, KR335, and KR300. For generating the LgBiT line, we used the pTEOE vector previously described and inserted the LgBiT amplicon at the XhoI site using in-fusion cloning^73^. The LgBiT ORF was amplified from the LgBiT expression vector from Promega using primers KR256 and KR258. For CRISPR/Cas9 editing, we used the Cas9-encoding pAIO3 plasmid, previously described^74^. Primers and the corresponding reverse complement needed to make gRNAs 45 were annealed in a thermal cycler and inserted into the AvrII restriction site of pAIO3 by In-Fusion cloning. All plasmids and their parasite integration products were analyzed by PCR and sequencing. A list of primers used in this study is provided as Supplementary Data 2. All primers were purchased from Integrated DNA Technologies.

### Parasite culture, transfection, selection, and synchronization

NF54 *attB* parasites expressing Cre recombinase^32,33^ were maintained in human red blood cells (3% hematocrit) prepared in RPMI 1640 supplemented with AlbuMAX (2.5 g/L), sodium pyruvate (110 mg/L), hypoxanthine (15 mg/L), HEPES (1.19 g/L), sodium bicarbonate (2.52 g/L), glucose (2 g/L), and gentamycin (10 μg/L). For CRISPR/Cas9 editing of the endogenous *pfgcn5* locus, we used cultures with more than 5% ring stages and transfected parasites via electroporation using a Bio-Rad Gene Pulser. For complementation of the PfGCN5^LoxP^ line, plasmids containing WT, GCN5 homologs, PyChimera or deletion constructs were independently co-transfected with a Bxb1 integrase plasmid for integration at the non-essential locus *cg6*^32^. For rapamycin-mediated excision, we used a concentration of 20 nM for 24 h. While the PfGCN5^LoxP^ line was maintained in 2.5 μg/mL blasticidin (BSD), all rescue lines were kept in a medium containing 2.5 μg/mL BSD and 5 nM WR99210 (WR). The LgBiT or LgBiT-HiBiT lines were supplemented with 12.5 μg/mL DSM1 or 12.5 μg/mL DSM1 and 5 nM WR, respectively, for selection. Parasites were cloned by limiting dilution seeding ~0.5 parasites per well in a 96-well plate. For synchronization, asynchronous cultures with more than 5% schizont stages were allowed to run through a MACS LD magnet column (Miltenyi Biotec). After a wash with warmed media, the column was removed from the magnet and eluted into a 15 mL conical tube. Purified schizonts were allowed to egress for 3 h in uninfected red blood cells (Supplementary Fig. 8). Newly infected red blood cells were purified by incubation with 5% sorbitol for 10 min.

### Flow cytometry and growth curves

Parasites were seeded at a starting parasitemia of 1% with or without rapamycin and plated in triplicate on a 96-well plate. Every other day, the media was changed, and the parasitemia was measured by flow cytometry using 50,000 events recorded per sample in an Attune NxT Flow cytometer. Supplementary Fig. 8 shows the gating strategies used in this study. Cells were stained with acridine orange diluted 1:25 in PBS. On day 4, after parasitemia was recorded, cultures were diluted 1:5 to prevent overgrowth. Cumulative parasitemia was back-calculated based on the dilution factor. Measured parasitemia on day 0 was subtracted from the final parasitemia obtained on day 6 to control for differences at the start of the experiment. The percentage growth for the rescue lines was calculated as the ratio between the average parasitemia of each line and the parasitemia of the PfGCN5^LoxP^ parasites in the absence of RAP on day 6, multiplied by 100.

### Western blotting

To visualize the PfGCN5 endogenous protein, we processed at least 5 mL of culture at 5% parasitemia and lysed it in PBS containing 0.035% saponin at 4 °C for 5 min. Saponin pellets were then resuspended in 1x sample buffer containing beta-mercaptoethanol and boiled at 99 °C. After centrifugation at 17,000×*g* at 4 °C for 10 min, a fraction of the supernatant was subjected to SDS-PAGE and immunoblotting. To visualize the GCN5 variants, we processed at least 20 mL of culture at 5% parasitemia and lysed as described above. Saponin pellets were resuspended in an NP40-containing lysis buffer (0.1% NP40, 50 mM Tris, and 150 mM NaCl, 1x HALT protease inhibitor) and subjected to three cycles of freezing and thawing, followed by sonication. After spinning at 17,000×*g* at 4 °C for 10 min, protein lysates were incubated with mouse anti-FLAG magnetic beads overnight at 4 °C. Elution was performed in boiling 2x sample buffer containing beta-mercaptoethanol. Flow-throughs were used to visualize various loading controls upon western blotting. Primary antibodies included rabbit anti-HA (1:1000), rat anti-FLAG (1:1000), mouse anti-histone H3 (1:1000), mouse anti-H3K9ac (1:1000), mouse anti-LgBiT (1:1000), mouse anti-HiBiT (1:1000), rabbit anti-HAD1 (1:1000), and mouse anti-PMV (1:500). In each case, corresponding IRDye conjugated secondary antibodies were used at 1:10,000 dilution. An Odyssey imaging system (Licor) was utilized to visualize blots. Uncropped and unprocessed scans of all the blots presented in this paper can be found in the Source Data file.

### Immunofluorescence assays

Cells were fixed and permeabilized in Hemacolor® fixing solution (product number 1.11955) for 10 s and then rinsed three times in PBS. Blocking was performed using 3% BSA in PBS for 1 h at room temperature or overnight at 4 °C. Dilutions used for primary antibodies are as follows: rabbit anti-HA (1:500), rat anti-FLAG (1:500), rabbit anti-aldolase (1:500), mouse anti-LgBiT (1:250), and mouse anti-HiBiT (1:500). Secondary antibodies, Alexa Fluor 488 or 555 (Life Technologies), were used at a 1:2000 dilution. ProLong antifade and 4’,6’-diamidino-2-phenylindole (DAPI) (Invitrogen) were used to mount cells. Images were taken in a Zeiss AxioImager M1 epifluorescence microscope with a Hamamatsu ORCA-ER CCD camera and the AxioVision software v. 4.8.1 using a 100x objective. Scale bars, cropping, and adjustments to brightness and contrast were made for display purposes with Zen Lite v. 3.12 (Zeiss).

### Cytoplasmic and nuclear fractionation

Parasites were lysed in half of the culture volume with cold 0.035% saponin for 4 min at 4 °C and washed with cold 1x PBS. Parasite pellets were then resuspended in cytoplasmic lysis buffer (25 mM Tris-HCl pH 7.5, 10 mM NaCl, 1.5 mM MgCl_2_, 1% Igepal, halt protease inhibitor cocktail, 1 mM PMSF, 50 mM sodium fluoride and 1 mM sodium orthovanadate) and incubated on ice for 30 min. For complete and gentle homogenization, samples were macerated in a cold glass douncer and centrifuged at 17,000×*g* for 10 min at 4 °C. Supernatants were saved as cytoplasmic fractions, and the remaining pellets were lysed in 0.1% Igepal buffer containing 150 mM NaCl and 50 mM Tris-HCl pH 7.6 for immunoprecipitations or 1x sample buffer containing beta-mercaptoethanol for western blots.

### Immunoprecipitation

Saponin pellets were resuspended in 0.1% Igepal buffer (150 mM NaCl and 50 mM Tris-HCl pH 7.6) containing Halt™ protease inhibitor cocktail EDTA-free (ThermoFisher, cat. number 78425), 1 mM AEBSF, 50 mM sodium fluoride and 1 mM sodium orthovanadate. After three cycles of freezing and thawing, samples were sonicated and spun at 13,500×*g* for 10 min at 4 °C. The recovered supernatant was incubated overnight at 4 °C with mouse-anti FLAG magnetic beads (Sigma, cat. number M8823) or mouse-anti HiBiT magnetic beads (cat. number CS3278A08). Antigen-conjugated beads were magnetized and washed two times with 1X TBS. The antigen was eluted in boiling 2X sample buffer containing beta-mercaptoethanol. In some cases, elution was performed using 0.1 M Glycine pH 2.0 in continuous rotation at room temperature for 15 min. Once the eluted antigen was recovered, the pH was neutralized using 1 M Tris pH 8.0.

### Histone extraction

Approximately 10 mL of synchronous cultures at 5% parasitemia and 3% hematocrit were treated with cold 0.035% saponin in PBS and then washed with cold PBS. Cell pellets were washed twice with a nuclear extraction buffer (15 mM Tris-HCl pH 7.5, 15 mM NaCl, 60 mM KCl, 5 mM MgCl_2_, 1 mM CaCl_2_, 250 mM sucrose, 500 μM AEBSF, 1 mM DTT, 5 nM microcystin, 10 mM sodium butyrate, and 1x HALT protease cocktail inhibitor). Briefly, washed cell pellets were treated with the above-described nuclear extraction buffer containing 0.3% NP-40 and incubated on ice for 30 min, followed by homogenization in a chilled douncer. After centrifugation at 2000×*g* at 4 °C for 10 min, the supernatant (cytosolic fraction) was removed, and the pellet (nuclear fraction) was washed twice in the nuclear extraction buffer. Following centrifugation as above, the pellet was resuspended in chilled 0.2 M H_2_SO_4_ and incubated with constant rotation for 2 h at 4 °C. Posterior to centrifugation at 3400×*g* at 4 °C for 10 min, solubilized histones were then treated with 100% trichloroacetic acid (~25% of the total volume) and incubated overnight at 4 °C. Precipitated histones were then washed with cold acetone containing 0.1% HCl, followed by a final wash with ice-cold acetone before resuspending extracted histones in 50 mM ammonium bicarbonate.

### Mass spectrometry identification of histone acetylation modifications

The histones were extracted and prepared for chemical derivatization and digestion as described previously^75,76^. In brief, the lysine residues from histones were derivatized with the propionylation reagent (1:2 reagent:sample ratio) containing acetonitrile and propionic anhydride (3:1), and the solution pH was adjusted to 8.0 using ammonium hydroxide. The propionylation was performed twice, and the samples were dried on speed vac. The derivatized histones were then digested with trypsin at a 1:50 ratio (wt/wt) in 50 mM ammonium bicarbonate buffer at 37 °C overnight. The N-termini of histone peptides were derivatized with the propionylation reagent twice and dried on speed vac. The peptides were desalted with the self-packed C18 stage tip. The purified peptides were then dried and reconstituted in 0.1% formic acid. An LC-MS/MS system, consisting of a Vanquish Neo UHPLC coupled to an Orbitrap Exploris 240 (Thermo Scientific), was used for peptide analysis. Histone peptide samples were maintained at 7 °C on a sample tray in LC. Separation of peptides was carried out on an Easy-Spray™ PepMap™ Neo nano-column (2 µm, C18, 75 µm × 150 mm) at room temperature with a mobile phase. The chromatography conditions consisted of a linear gradient from 2 to 32% solvent B (0.1% formic acid in 100% acetonitrile) in solvent A (0.1% formic acid in water) over 48 min and then 42 to 98% solvent B over 12 min at a flow rate of 300 nL/min. The mass spectrometer was programmed for data-independent acquisition (DIA). One acquisition cycle consisted of a full MS scan, 35 DIA MS/MS scans of 24 m/z isolation width starting from 295 m/z to reach 1100 m/z. Typically, full MS scans were acquired in the Orbitrap mass analyzer across 290–1100 m/z at a resolution of 60,000 in positive profile mode with an auto maximum injection time and an AGC target of 300%. MS/MS data from HCD fragmentation were collected in the Orbitrap. These scans typically used an NCE of 30, an AGC target of 1000%, and a maximum injection time of 60 ms. Histone MS data were analyzed with EpiProfile^77^.

### HAT in vitro assay

PfGCN5 proteins were pulled down using mouse-anti FLAG magnetic beads, from at least 80 mL of culture at 3% hematocrit and 5% parasitemia. To washed antigen-conjugated beads, 2x HAT buffer (50 mM Tris-HCl pH 8.0, 10% glycerol, 1 mM dithiothreitol, 0.1 mM, and 1 mM of AEBSF), 10 μg of calf thymus, 10 mM butyric acid, and 60 μM acetyl-CoA or water were added. The solution was gently mixed with a pipette and incubated at 30 °C for 1 h with occasional mixing. To stop the reaction, 1x sample buffer containing β-mercaptoethanol was added and boiled for 5 min. The beads were magnetized and the supernatant was saved for analysis. About ¼ and ½ of the volume from each reaction was used to be analyzed in H3K9ac and FLAG blots, respectively.

### HiBiT blotting

Proteins were transferred to PDVF membranes, followed by 5 washes with 0.1% TBS-T. Wash buffer was discarded, and a solution containing the LgBiT protein diluted 200-fold in the 1X Nano-Glo® buffer was added and incubated overnight at 4 °C with gentle rocking. The next day, after allowing membranes to equilibrate to room temperature, furimazine (substrate) was added at a 1:500 dilution and incubated for 5 min before imaging.

### Luminescence assays

Live-cell kinetics of cytosolic HiBiT-tagged fragments were determined using the Nano-Glo® Live Cell Assay System (Promega, cat. number N2011) as per manufacturer instructions. Briefly, 100 μL of cell cultures at 3% hematocrit and 5% parasitemia were mixed with 25 μL of the Nano-Glo® buffer containing the substrate at 1:20 dilution in opaque white 96-well plates. After gently mixing by hand, luminescence was immediately measured in a Perkin Elmer EnVision 2103 microplate reader. Total levels of HiBiT-tagged fragments were determined using the Nano-Glo® HiBiT Lytic Detection System (Promega, cat. number N3030) as per manufacturer instructions. An equal volume of cell cultures at 3% hematocrit and 5% parasitemia was mixed with the lytic buffer containing the LgBiT protein and substrate at a 1:100:50 dilution. Plates were incubated in a dark environment at room temperature for 10 min before measuring luminescence in a Perkin Elmer EnVision 2103 microplate reader.

### Statistical analysis

While some figures display mean values with error bars representing standard deviations or the standard error of the mean from three or four independent biological replicates, others show the distribution of three or four independent biological replicates each with three technical replicates. In the latter, the third and first quartile and the median are represented. Significant differences were evaluated by the two-tailed Student’s *t*-test or by one-way ANOVA with Tukey’s or Fisher’s multiple comparisons post hoc test using GraphPad Prism 11.0.0. Western blots were analyzed by densitometry using Image Studio Lite 5.2. All graphs were prepared using GraphPad Prism 11.0.0.

### Reporting summary

Further information on research design is available in the Nature Portfolio Reporting Summary linked to this article.

## Supplementary information

Supplementary Information

Description Of Additional Supplementary File

Supplementary Data 1

Supplementary Data 2

Reporting summary

Transparent Peer Review file

## Source data

Source Data

## Data Availability

The authors confirm that all relevant data are included here and/or in the supplementary information. Data used to generate each graph and uncropped gels or blots are provided as a Source Data file in a Microsoft Excel format. GCN5 sequence homology conservation across organisms and *Plasmodium* spp. is shown in Supplementary Figs. 1 and 2. Plasmid maps and cloning strategies are shown in Supplementary Figs. 3–6. Information for LC-MS/MS from C-terminal FLAG pull downs are summarized in Supplementary Table 1. The entire list of pulled-down proteins can be found in Supplementary Data 1. Sequence coverage of PfGCN5 across deletion proteins is shown in Supplementary Fig. 7. The histone acetylation proteomic data generated in this study have been uploaded to MassIVE with identifiers MSV000099212 and PXD068464 [https://massive.ucsd.edu/ProteoSAFe/dataset.jsp?task=aad3332d2158402bb4a7d10724dae948]. The list of primers used in this study is presented as Supplementary Data 2. Source data are provided with this paper.
