# Supplementary material for: A role for the poly-asparagine repeat in the Plasmodium histone acetyltransferase, PfGCN5
Source: Nat Commun. 2026 Jun 20;17:7799. doi: 10.1038/s41467-026-74632-6 (PMC13438671; doi:10.1038/s41467-026-74632-6)
Supplement: Supplementary file 1 [file 41467_2026_74632_MOESM1_ESM.pdf]

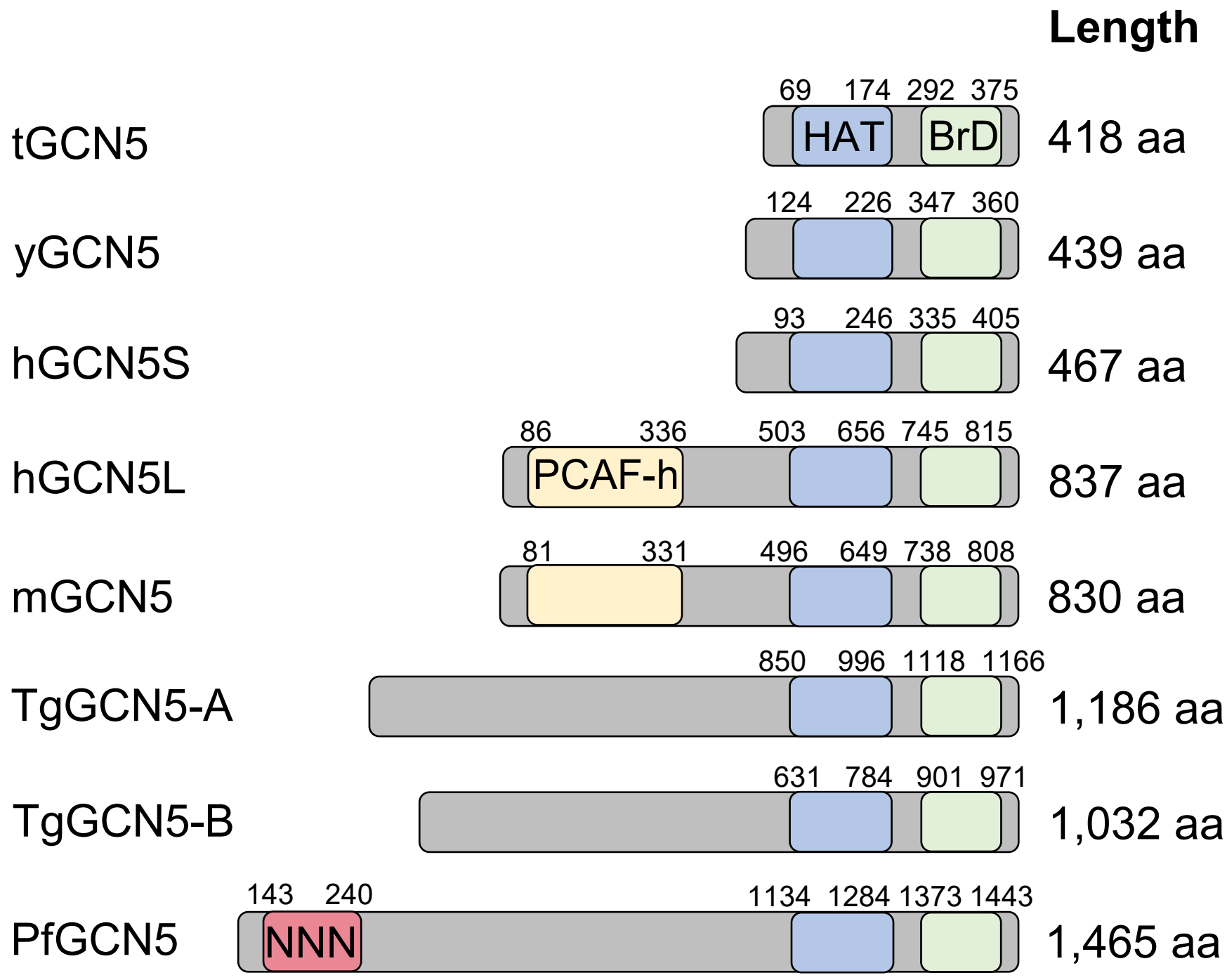

Supplementary Fig. 1. Conservation of functional domains in GCN5. Comparative diagram of GCN5 homologs from *Tetrahymena thermophila* (tGCN5), *Saccharomyces cerevisiae* (yGCN5), *Homo sapiens* (hGCN5S: short isoform ; hGCN5L: long isoform), *Mus musculus* (mGCN5), *Toxoplasma gondii* (TgGCN5A/B), and *Plasmodium falciparum* (PfGCN5). The P300/CBP-associated factor (PCAF)-homology domain is shown in yellow, the histone acetyltransferase (HAT) domain in blue, the bromodomain (BrD) in green, and the poly-N repeat in pink. Numbers above each domain/motif indicate the start and the end of such region according to data collected from UniProt using the PROSITE database.

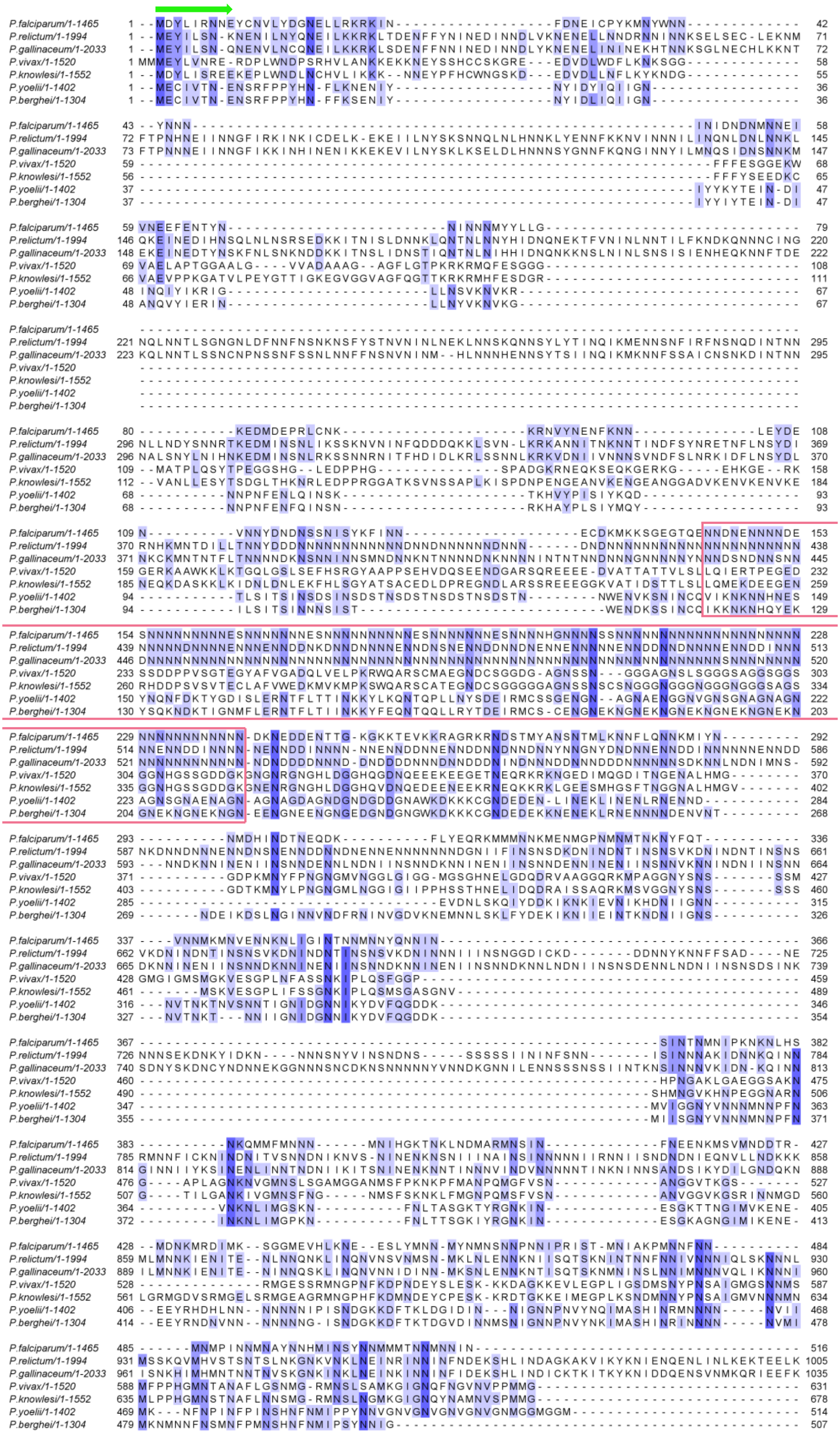

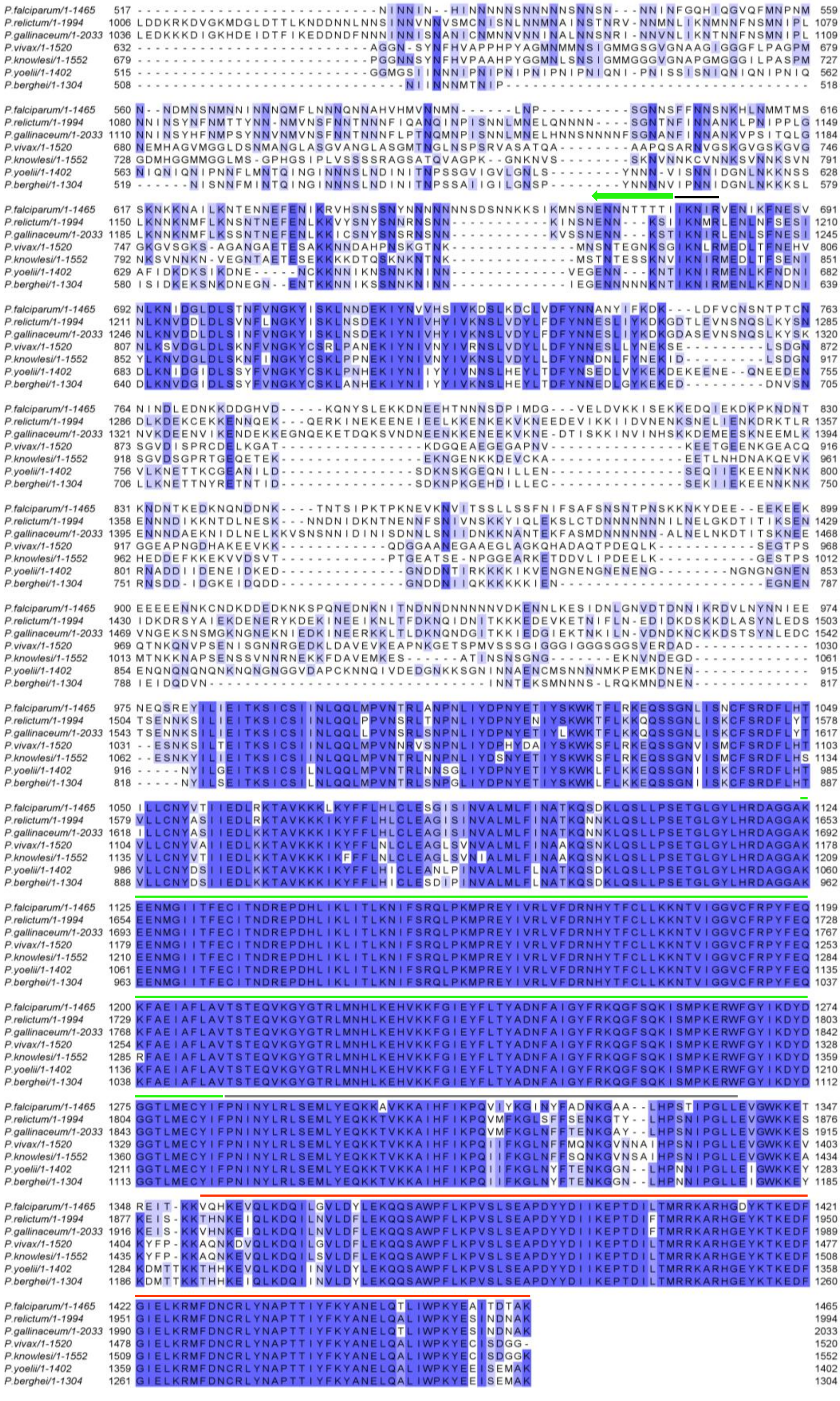

Supplementary Fig. 2. Multiple sequence alignment analysis across plasmodial GCN5 homologs using Clustal Omega and visualized in Jalview. The region flanked by the two green arrows depicts the PfGCN5 N terminus region used to make the PyGCN5 chimeric construct. The black line highlights the conserved motif IKNI/M/LR at amino acid position 677 used to define the PfGCN5 N terminus in the chimeric construct. Regions in the pink box correspond to the poly-N or GS-repeat. Green, gray, and red lines indicate the HAT, putative ADA2-binding, and bromo domains.

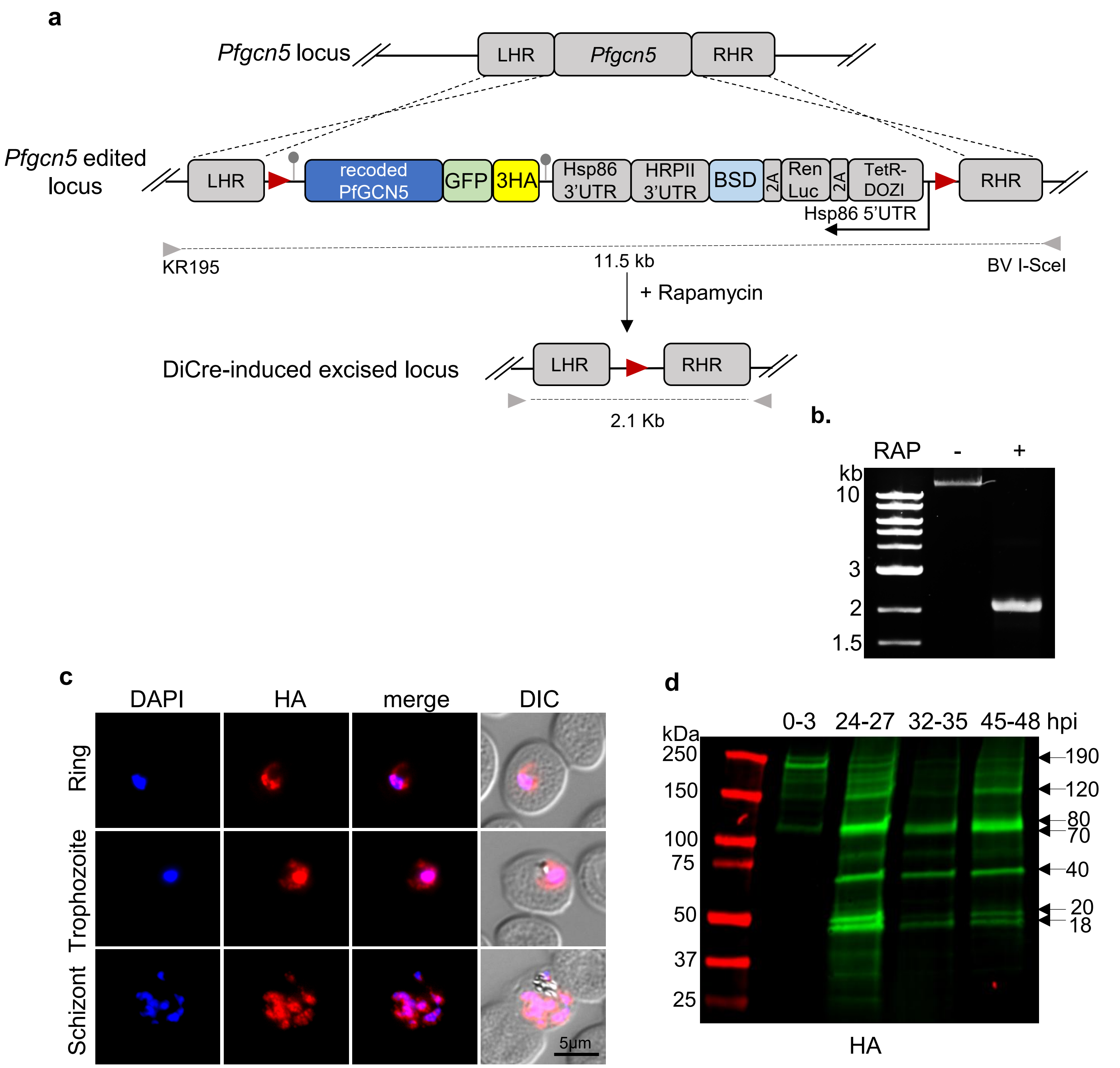

Supplementary Fig. 3. Generation and characterization of the PfGCN5^{LoxP} line. a) edited *pfgcn5* locus showing LoxP sites (red triangles) and regulatory elements: the native promoter embedded in the left homologous region (LHR), Hsp86 3'UTR stabilizing the *pfgcn5* transcript, Hsp86 5'UTR and HRPII 3' UTR driving expression and stabilizing transcript from the TetR-DOZI fusion protein, *Renilla* luciferase, and the resistance cassette, blasticidin (BSD), respectively. A *Saccharomyces cerevisiae* recoded *pfgcn5* coding sequence was C-terminally tagged with GFP (green) and 3xHA (yellow). Both the LHR and the right homologous region (RHR) were used for homologous recombination using CRISPR/Cas9. Gray arrowheads represent primers KR195 and BV I-SceI used to check integration and rapamycin-mediated excision. Gray lollipops represent aptamers. **b)** Agarose gel stained with ethidium bromide showing excision of the entire *pfgcn5* locus 30 hours after adding rapamycin. Expected bands of 11.5 kb (-RAP) and 2.1 kb (+RAP) are observed **c)** Immunofluorescence analysis of PfGCN5 localization using anti-HA antibodies. DAPI stain was used for nuclear visualization. **d)** Representative western blot showing the processing of PfGCN5 throughout the intraerythrocytic cycle. Parasites were synchronized by magnet purification of late schizonts followed by sorbitol treatment 3 hours later. Arrows indicate the molecular mass (kDa) after subtracting the mass of the GFP tag.

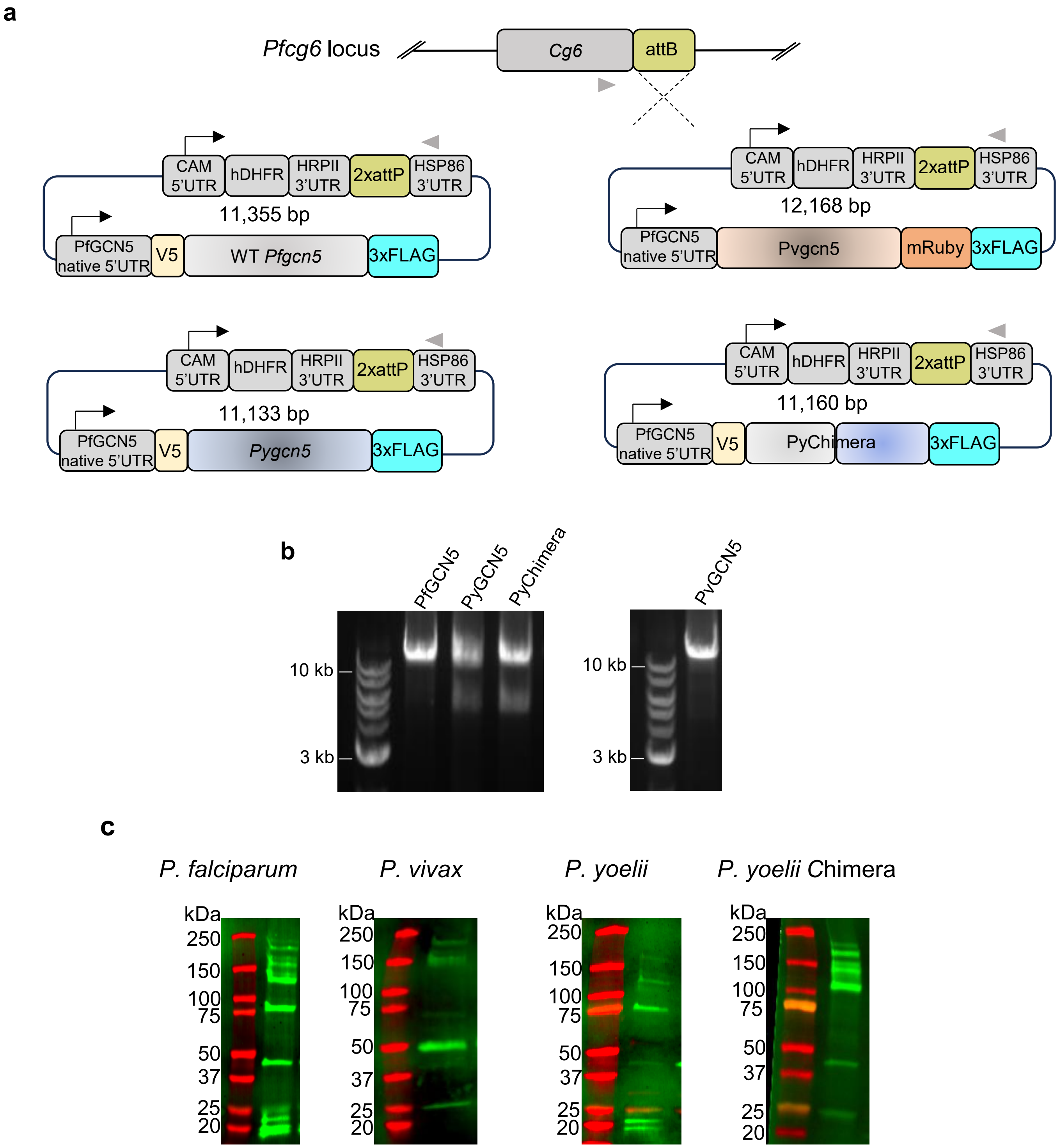

Supplementary Fig. 4. Generation and validation of the WT, homologs and PyChimera rescue lines. a) Our pEOE-hDHFR-2xattP plasmid was modified to encode the native *pfgcn5* promoter and the WT *Pfgcn5, or Pvgcn5 or Pygcn5* or PyChimera coding sequence. Integration catalyzed by the mycobacteriophage Bxb1 serine integrase occurs between a genome-encoded *attB* site in the non-essential *cg6* gene and the plasmid-encoded *attP* site, resulting in the integration of the entire plasmid. **b)** Correct integration was verified by sequencing of an amplicon generated with the attB_Fw primer sitting on the genomic region upstream of the *attB* site and the attP_Rv primer sitting in the HSP86 3'utr of the plasmid (primers are depicted by gray arrowheads). Shown are the amplicons run on a 1% agarose gel. **c)** Identification of the second WT copy, and GCN5 variant proteins, expressed under the control of the native *pfgcn5* promoter, through western blotting analysis using anti-FLAG antibodies. Visualization of V5-tagged fragments failed likely due to cleavage.

a

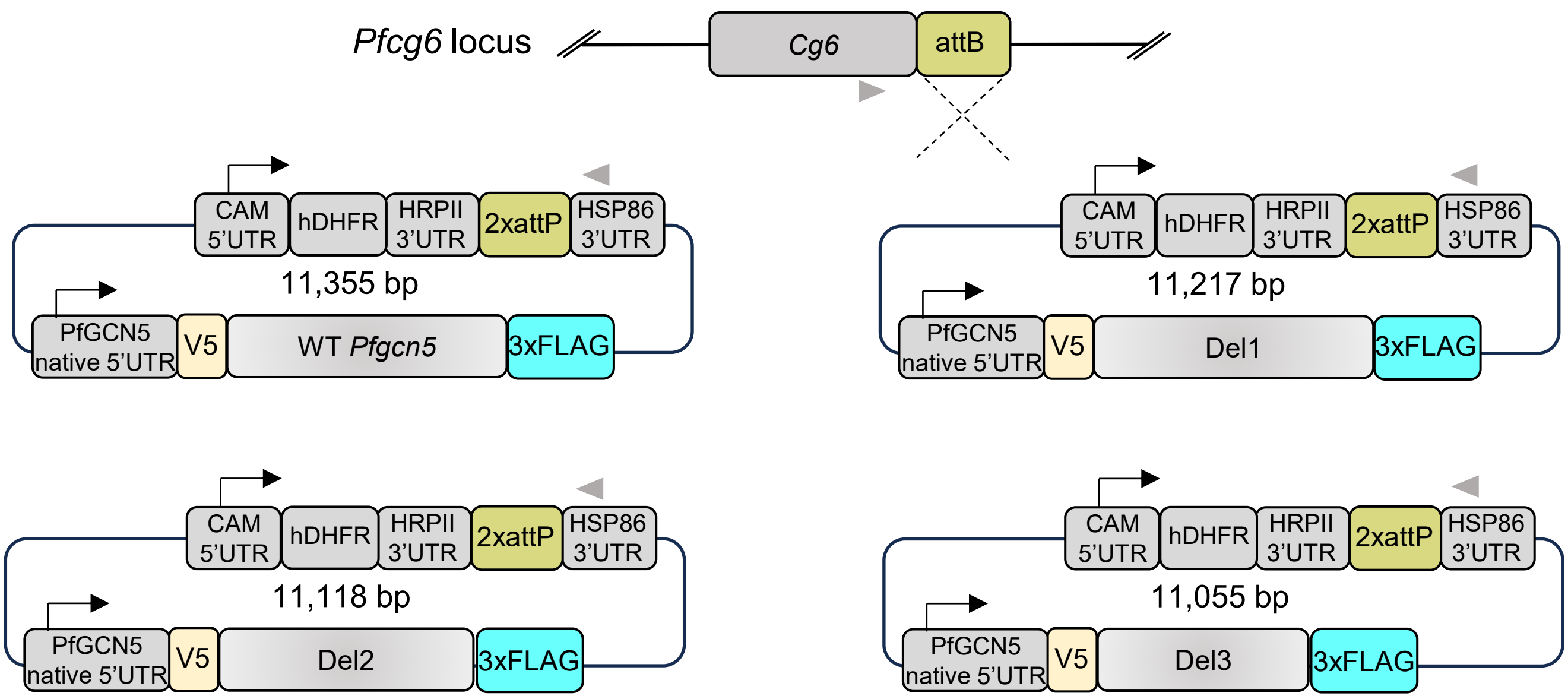

b

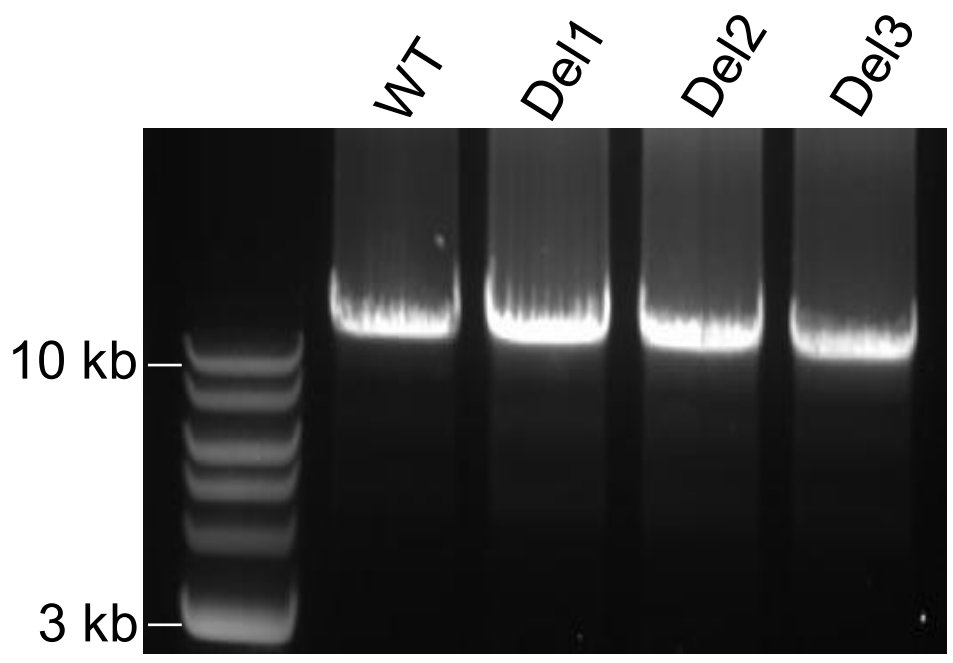

Supplementary Fig. 5. Generation of the deletion rescue lines. a) Our pEOE-hDHFR-2xattP plasmid was modified to encode the native *pfgcn5* promoter and the WT *pfgcn5* coding sequence or incremental deletions in the poly-N repeat from the C-terminal end. Deletion #1 (Del1) retains 48 N, deletion #2 (Del2) 23 N, and deletion #3 (Del3) 7 N in comparison to 81N found in the WT protein. Integration catalyzed by the mycobacteriophage Bxb1 serine integrase occurs between a genome-encoded *attB* site in the non-essential *cg6* gene and the plasmid-encoded *attP* site, resulting in the integration of the entire plasmid. **b)** Correct integration was verified by sequencing of an amplicon generated with the attB_Fw primer sitting on the genomic region upstream of the *attB* site and the attP_Rv primer sitting in the HSP86 3'utr of the plasmid (primers are depicted by gray arrowheads). Shown are the amplicons run on a 1% agarose gel.

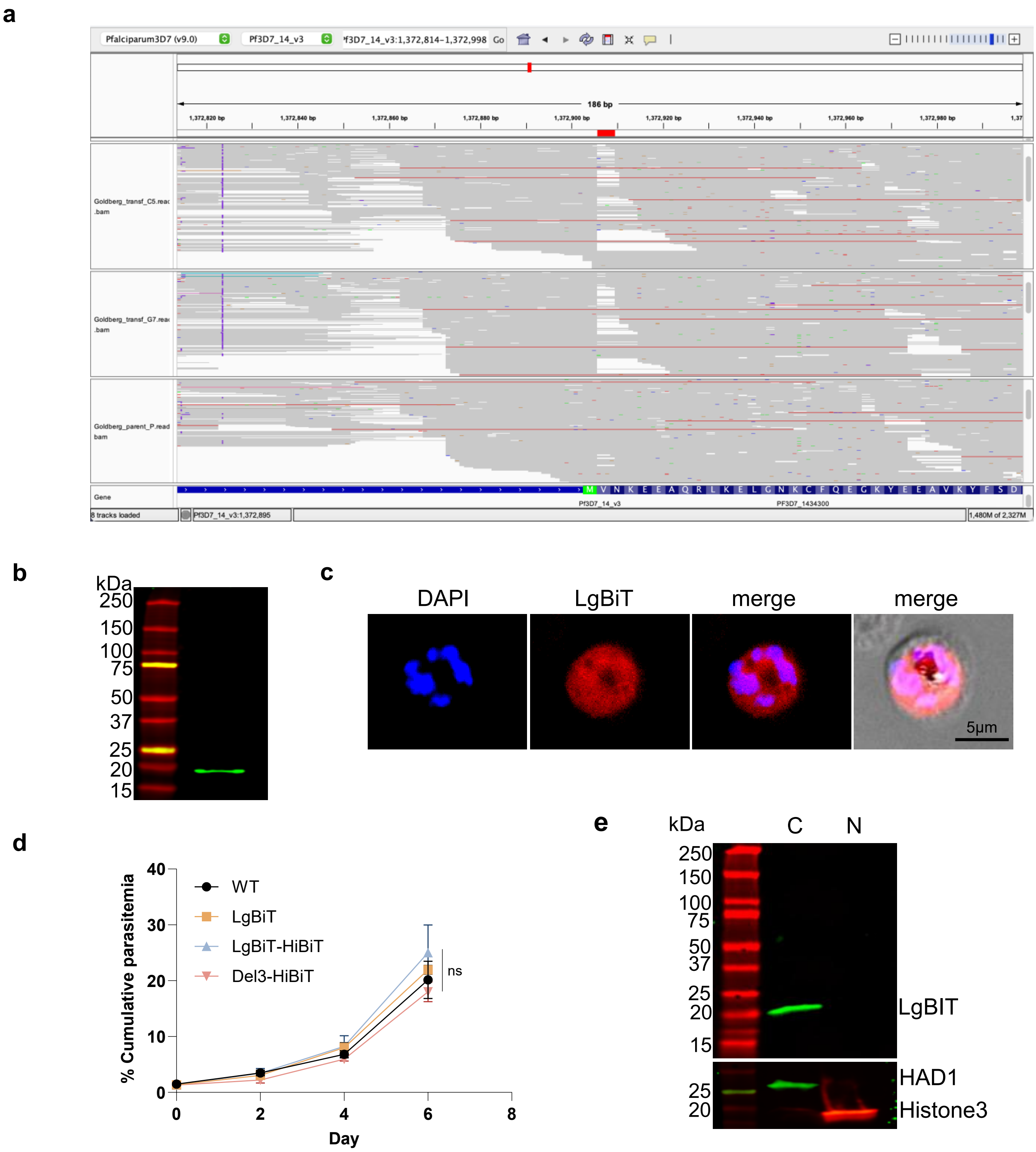

Supplementary Fig. 6. Characterization of the LgBiT-HiBiT line. a) Visualization of the aligned sequencing reads in the Integrative Genomics Viewer, showing insertion of LgBiT at position 1,372,910 bp of chromosome 14 in the two clones but not in the parental line. **b)** Representative western blot showing the expression of LgBiT using an anti-LgBiT specific antibody. **c)** Inspection of the cellular localization of the LgBiT protein using immunofluorescence analysis. **d)** Growth phenotype of the LgBiT, LgBiT-HiBiT, and Del3-HiBiT lines in comparison to the WT line assessed by flow cytometry every cycle for 6 days. Mean values from three independent experiments are shown, and error bars represent standard deviations. Data were analyzed statistically by a two-tailed Student's test. **e)** Cellular fractionation of the LgBiT-HiBiT line. LgBiT was identified using an anti-LgBiT antibody. Anti-HAD1 and anti-Histone 3 antibodies were used as markers of the cytosolic "C" or nuclear "N" fraction, respectively. The bottom blot was run separately and was used as an assessment of the fractionation.

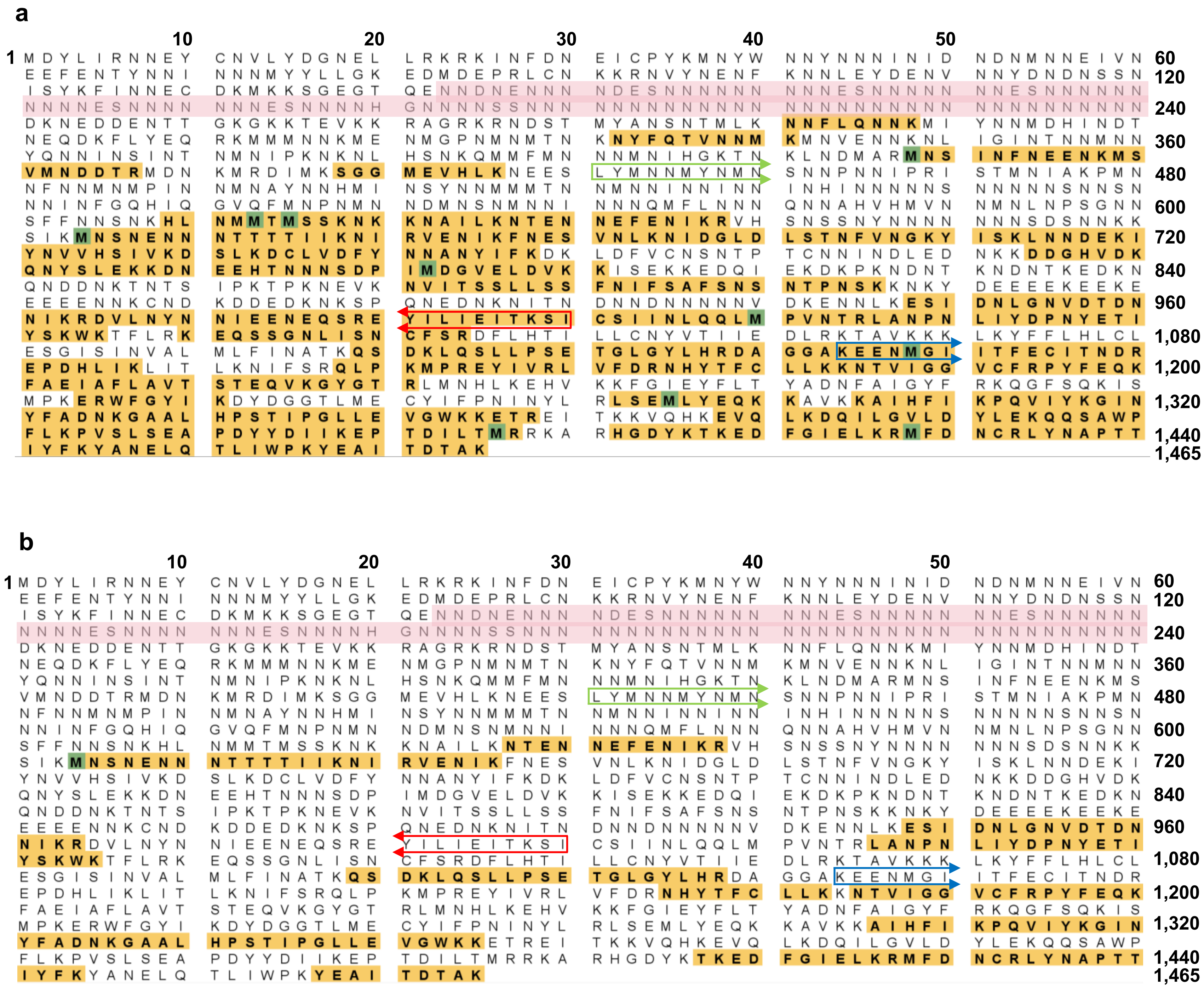
a
b

c

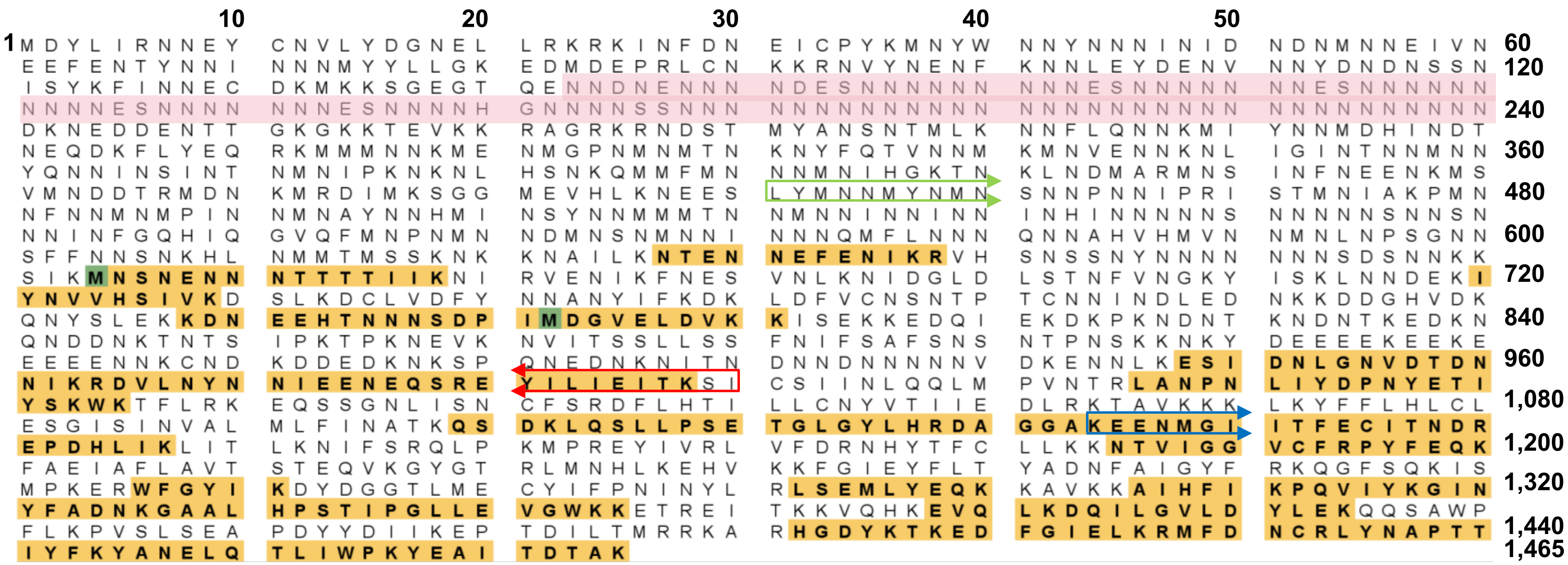

10 20 30 40 50
1 MDYLIRNNEY CNVLYDGNEL LRKRKINFDN EICPYKMNYW NNYNNNINID NDNMNNEIVN 60
EEFENTYNNI NNNMYYLLGK EDMDEPRLCN KKRNVYNENF KNNLEYDENV NNYDNDNSSN 120
ISYKFINNEC DKMKKSGEGT QENNDNENNN NDESNNNNNN NNNESNNNNN NNESNNNNNN
NNNNESNNNN NNNESNNNNH GNNNNSSNNN NNNNNNNNNN NNNNNNNNNN NNNNNNNNNN 240
DKNEDDENTT GKGKKTEVKK RAGRKRNDST MYANSNTMLK NNFLQNNKMI YNNMDHINDT
NEQDKFLYEQ RKMMMNNKME NMGPNMNMTN KNYFQTVNNM KMNVENNKNL IGINTNNMNN 360
YQNNINSINT NMNIPKNKNL HSNKQMMFMN NNMNIHGKTN KLNDMARMNS INFNEENKMS
VMNDDTRMDN KMRDIMKSGG MEVHLKNEES LYMNNMYNMN SNNPNNIPRI STMNIAKPMN 480
NFNNMNMPIN NMNAYNNHMI NSYNNMMMTN NMNNINNINN INHINNNNNS NNNNNSNNSN
NNINFGQHIQ GVQFMNPNMN NDMNSNMNNI NNNQMFLNNN QNNAHVHMVN NMNLNPSGNN 600
SFFNNSNKHL NMMTMSSKNK KNAILKNTEN NEFENIKRVH SNSSNYNNNN NNNSDSNNKK
SIKMNSNENN NTTTTIIKNI RVENIKFNES VNLKNIDGLD LSTNFVNGKY ISKLNNDEKI 720
YNVVHSIVKD SLKDCLVDFY NNANYIFKDK LDFVCNSNTP TCNNINDLED NKKDDGHVDK
QNYSLEKKDN EEHTNNNSDP IMDGVELDVK KISEKKEDQI EKDKPKNDNT KNDNTKEDKN 840
QNDDNKTNTS IPKTPKNEVK NVITSSLLSS FNIFSAFSNS NTPNSKKNKY DEEEEKEEKE
EEEENNKCND KDDEDKNKSP QNEDNKNITN DNNDNNNNNV DKENNLKESI DNLGNVDTDN 960
NIKRDVLNYN NIEENEQSRE YILIEITKSI CSIINLQQLM PVNTRLANPN LIYDPNYETI
YSKWKTFLRK EQSSGNLISN CFSRDFLHTI LLCNYVTIIE DLRKTAVKKK LKYFFLHLCL 1,080
ESGISINVAL MLFINATKQS DKLQSLLPSE TGLGYLHRDA GGAKEENMGI ITFECITNDR
EPDHLIKLIT LKNIFSRQLP KMPREYIVRL VFDRNHYTFC LLKKNTVIGG VCFRPYFEQK 1,200
FAEIAFLAVT STEQVKGYGT RLMNHLKEHV KKFGIEYFLT YADNFAIGYF RKQGFSQKIS
MPKERWFGYI KDYDGGTLME CYIFPNINYL RLSEMLYEQK KAVKKAIHFI KPQVIYKGIN 1,320
YFADNKGAAL HPSTIPGLLE VGWKKETREI TKKVQHKEVQ LKDQILGVLD YLEKQQSAWP
FLKPVSLSEA PDYYDIIKEP TDILTMRRKA RHGDYKTKED FGIELKRMFD NCRLYNAPTT 1,440
IYFKYANELQ TLIWPKYEAI TDTAK 1,465

d

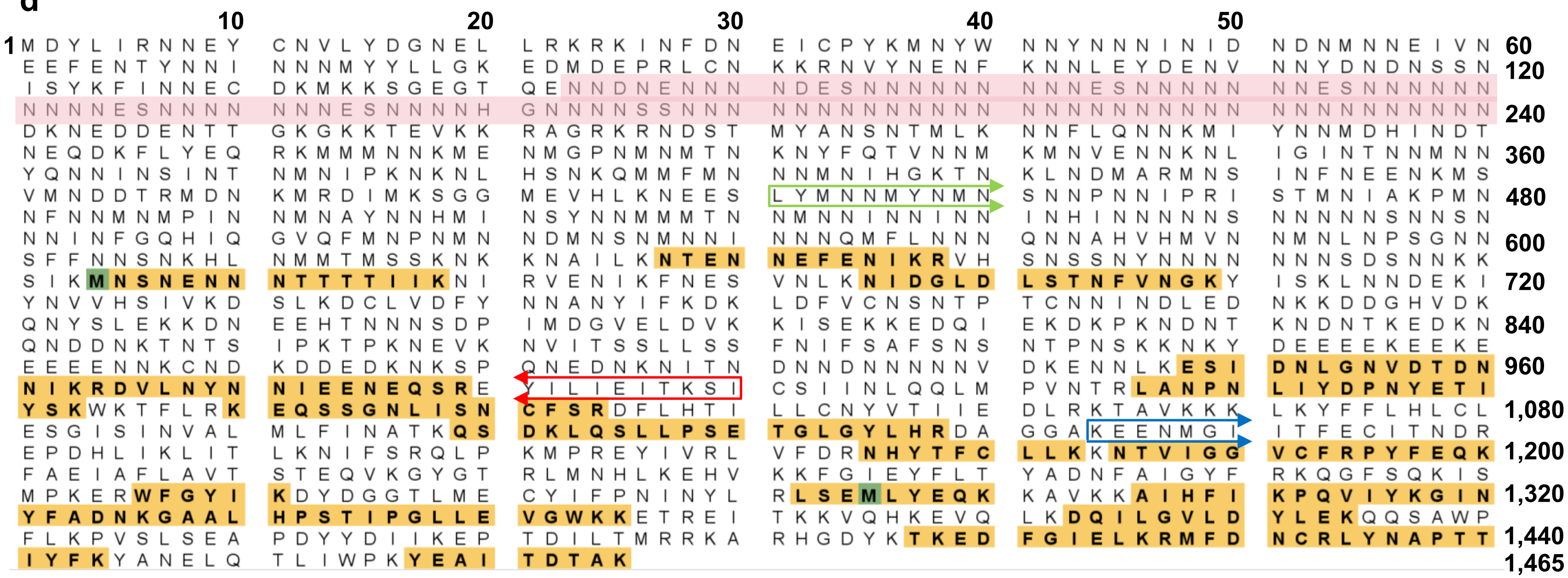

10 20 30 40 50
1 MDYLIRNNEY CNVLYDGNEL LRKRKINFDN EICPYKMNYW NNYNNNINID NDNMNNEIVN 60
EEFENTYNNI NNNMYYLLGK EDMDEPRLCN KKRNVYNENF KNNLEYDENV NNYDNDNSSN 120
ISYKFINNEC DKMKKSGEGT QENNDNENNN NDESNNNNNN NNNESNNNNN NNESNNNNNN
NNNNESNNNN NNNESNNNNH GNNNNSSNNN NNNNNNNNNN NNNNNNNNNN NNNNNNNNNN 240
DKNEDDENTT GKGKKTEVKK RAGRKRNDST MYANSNTMLK NNFLQNNKMI YNNMDHINDT
NEQDKFLYEQ RKMMMNNKME NMGPNMNMTN KNYFQTVNNM KMNVENNKNL IGINTNNMNN 360
YQNNINSINT NMNIPKNKNL HSNKQMMFMN NNMNIHGKTN KLNDMARMNS INFNEENKMS
VMNDDTRMDN KMRDIMKSGG MEVHLKNEES LYMNNMYNMN SNNPNNIPRI STMNIAKPMN 480
NFNNMNMPIN NMNAYNNHMI NSYNNMMMTN NMNNINNINN INHINNNNNS NNNNNSNNSN
NNINFGQHIQ GVQFMNPNMN NDMNSNMNNI NNNQMFLNNN QNNAHVHMVN NMNLNPSGNN 600
SFFNNSNKHL NMMTMSSKNK KNAILKNTEN NEFENIKRVH SNSSNYNNNN NNNSDSNNKK
SIKMNSNENN NTTTTIIKNI RVENIKFNES VNLKNIDGLD LSTNFVNGKY ISKLNNDEKI 720
YNVVHSIVKD SLKDCLVDFY NNANYIFKDK LDFVCNSNTP TCNNINDLED NKKDDGHVDK
QNYSLEKKDN EEHTNNNSDP IMDGVELDVK KISEKKEDQI EKDKPKNDNT KNDNTKEDKN 840
QNDDNKTNTS IPKTPKNEVK NVITSSLLSS FNIFSAFSNS NTPNSKKNKY DEEEEKEEKE
EEEENNKCND KDDEDKNKSP QNEDNKNITN DNNDNNNNNV DKENNLKESI DNLGNVDTDN 960
NIKRDVLNYN NIEENEQSRE YILIEITKSI CSIINLQQLM PVNTRLANPN LIYDPNYETI
YSKWKTFLRK EQSSGNLISN CFSRDFLHTI LLCNYVTIIE DLRKTAVKKK LKYFFLHLCL 1,080
ESGISINVAL MLFINATKQS DKLQSLLPSE TGLGYLHRDA GGAKEENMGI ITFECITNDR
EPDHLIKLIT LKNIFSRQLP KMPREYIVRL VFDRNHYTFC LLKKNTVIGG VCFRPYFEQK 1,200
FAEIAFLAVT STEQVKGYGT RLMNHLKEHV KKFGIEYFLT YADNFAIGYF RKQGFSQKIS
MPKERWFGYI KDYDGGTLME CYIFPNINYL RLSEMLYEQK KAVKKAIHFI KPQVIYKGIN 1,320
YFADNKGAAL HPSTIPGLLE VGWKKETREI TKKVQHKEVQ LKDQILGVLD YLEKQQSAWP
FLKPVSLSEA PDYYDIIKEP TDILTMRRKA RHGDYKTKED FGIELKRMFD NCRLYNAPTT 1,440
IYFKYANELQ TLIWPKYEAI TDTAK 1,465

Supplementary Fig. 7. Sequence coverage from C-terminal pull-downs in the a) WT, b) Del1, c) Del2, and d) Del3 rescue lines. Peptides identified by mass spectrometry are shown in yellow, methionine residues susceptible to oxidation are shown in green and the poly-N repeat is shown in light pink. The largest N-terminal polypeptide (~120 kDa) is predicted to end at the sequence enclosed with the red arrows, and the largest C-terminal polypeptide (~120 kDa) is predicted to end at the sequence enclosed with the green arrows. The predicted start site for the HAT domain is enclosed with the blue arrows. Numbers indicate the amino acid position. Sequence coverage was visualized using the proteome software, Scaffold 5..

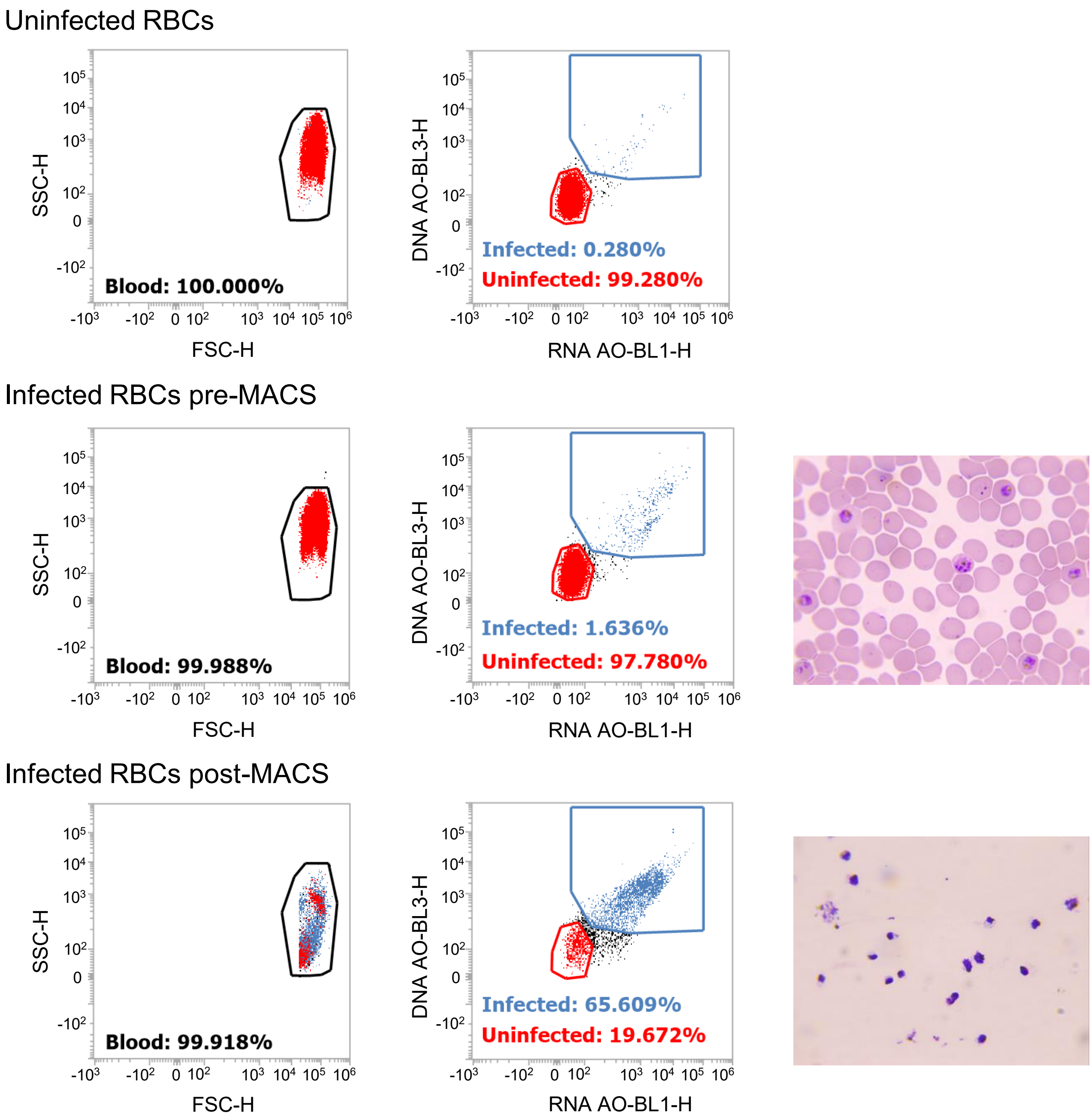

Supplementary Fig. 8. Gating strategy for determining parasitemia as described in Fig. 2b, 2e, Fig. 3c, and Fig. 4. Acridine orange-stained uninfected erythrocytes were gated (Blood, black) based on the forward scatter (FSC-H) and side scatter (SSC-H) of incident light (top, left). A total of 50,000 events within the blood population were then analyzed in the BL1-H and BL3-H channels (top, right). The uninfected (Uninfected, red) and infected (Infected, blue) populations were then gated. An acridine orange-stained sample of *P. falciparum* infected RBCs (hema-stained image on right, middle panel) showed two distinct populations on a BL1-H vs BL3-H dot plot. Following synchronization using MACS LD magnetic columns (Miltenyi Biotec), samples were analyzed under the same parameters (lower panel). As shown in the hema-stained image on bottom-right, the MACS purified sample showed enrichment of schizonts in the infected gate. The percentage of events in the infected gate was counted for parasite growth assays presented in the manuscript.

Complex	Protein name	Protein ID	Spectral counts				Normalized to GCN5				Fold change ± SD
			WT	Del1	Del2	Del3	WT	Del1	Del2	Del3	
SAGA-like	GCN5	PF3D7_0823300	99	18	29	23	1	1	1	1	N/A
	ADA2	PF3D7_1014600	35	12	13	8	0.35	0.67	0.45	0.35	1.4 ± 0.37
	PHD1	PF3D7_1008100	64	22	30	16	0.65	1.22	1.03	0.70	1.5 ± 0.3
	PHD2	PF3D7_1433400	38	7	4	3	0.38	0.39	0.14	0.13	0.6 ± 0.3
	NAPS	PF3D7_0919000	36	25	33	16	0.36	1.39	1.14	0.70	3 ± 0.8
Unknown	PHD finger protein	PF3D7_0310200	8	8	9	24	0.08	0.44	0.31	1.04	7.4 ± 3.9
	14-3-3I	PF3D7_0818200	22	49	31	37	0.22	2.72	1.07	1.61	8.1 ± 3.1

Supplementary table 1. Proteins interacting with PfGCN5 identified in C-terminal pull-downs couple with mass spectrometry. Spectral counts were normalized to that of GCN5 for each line. Fold change corresponds to the ratio between the average of normalized values across the deletion lines over that of the WT rescue line. SD: standard deviation. Values shown correspond to one biological replicate. The complete list of proteins can be found in the Supplementary Data 1 file.

Raw data for Supplementary blots/gels

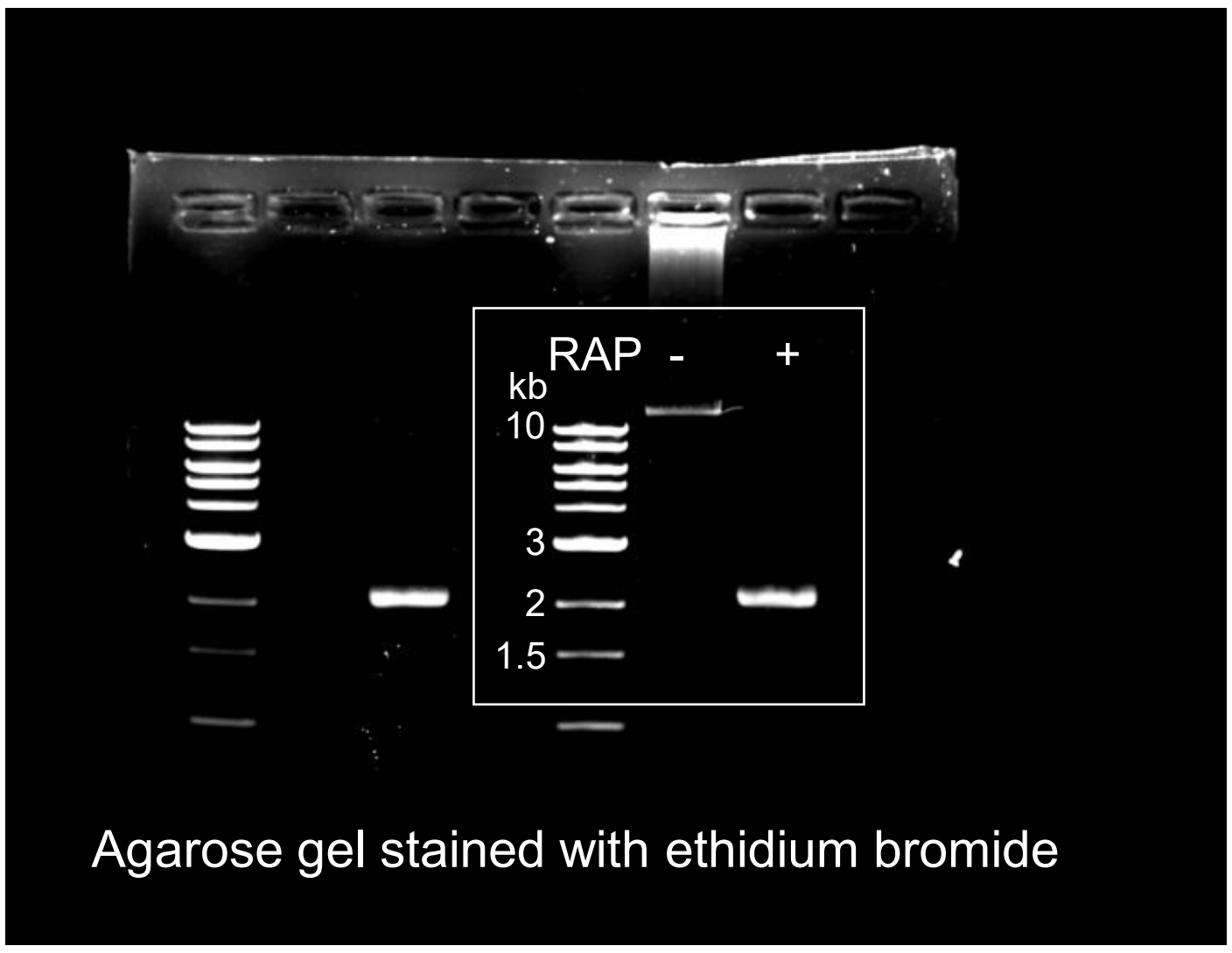

Supplementary Figure 3b

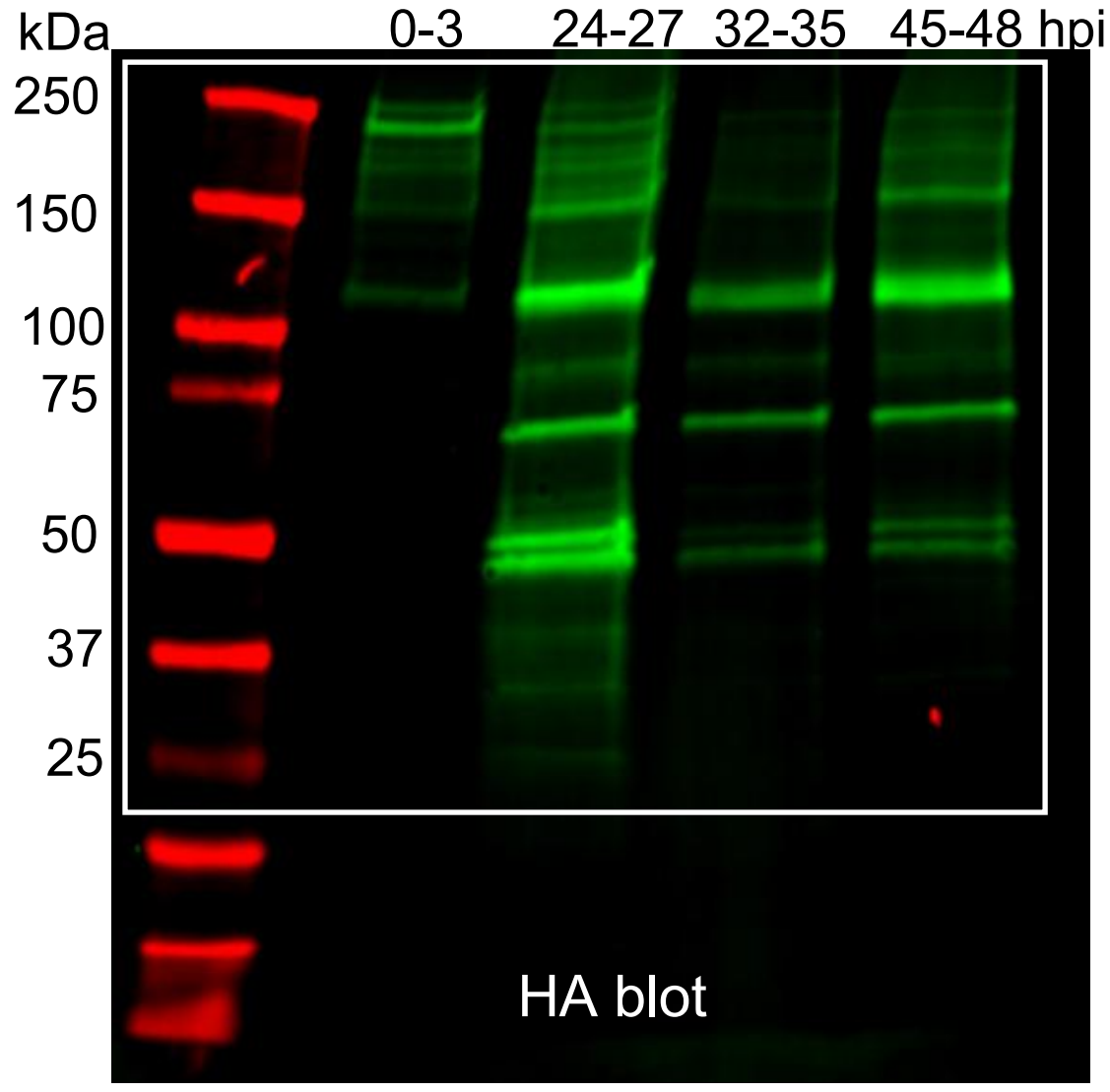

Supplementary Figure 3d

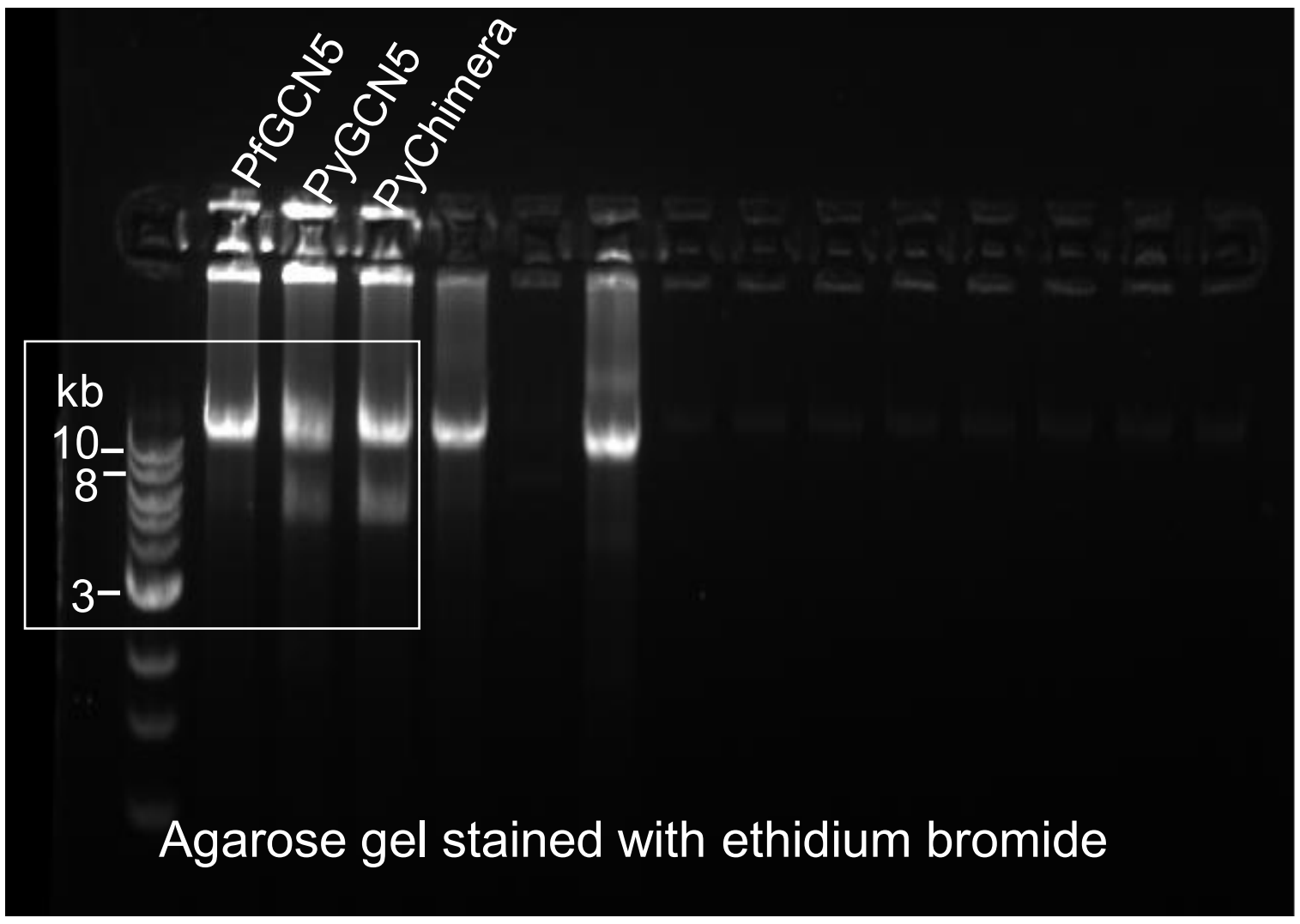

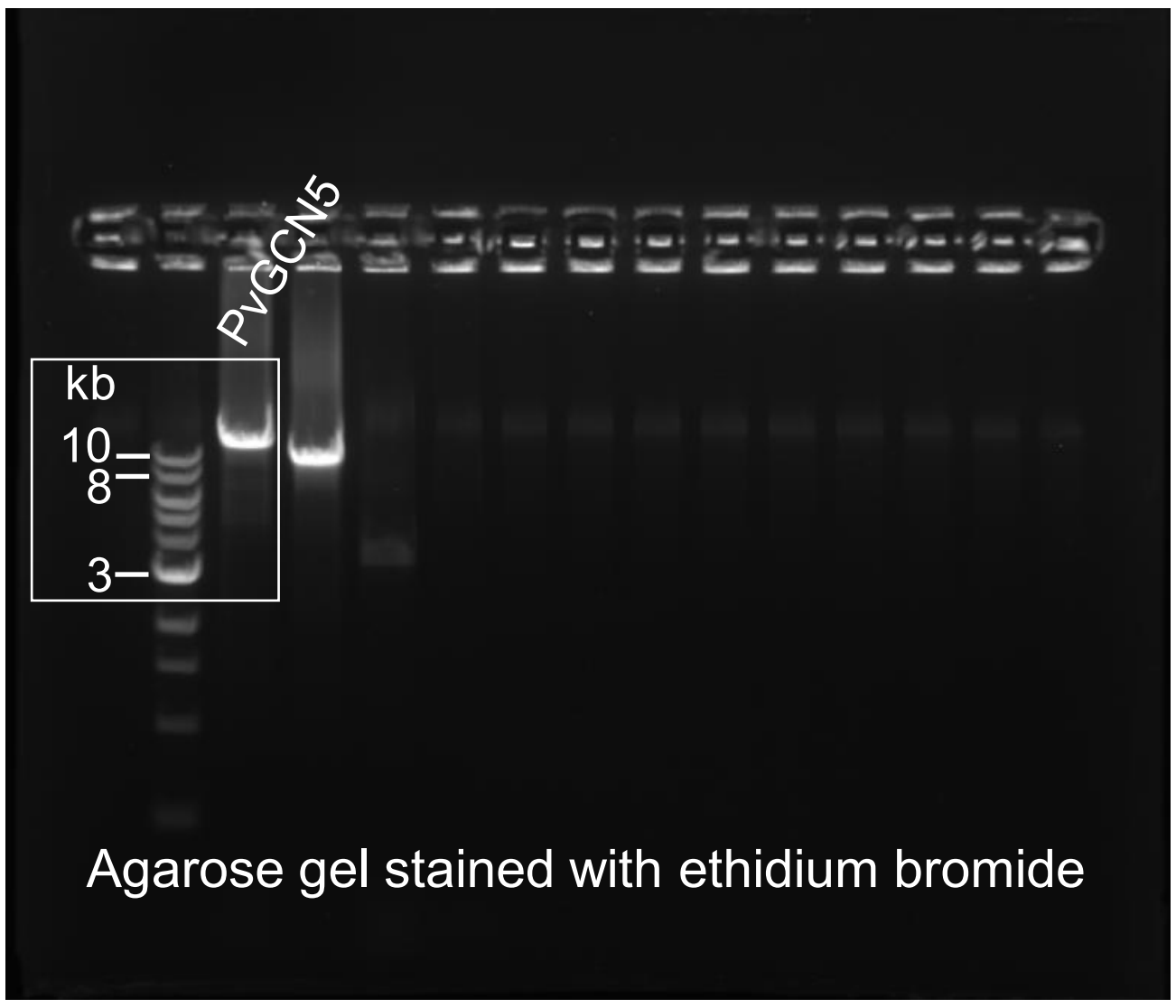

Supplementary Figure 4b

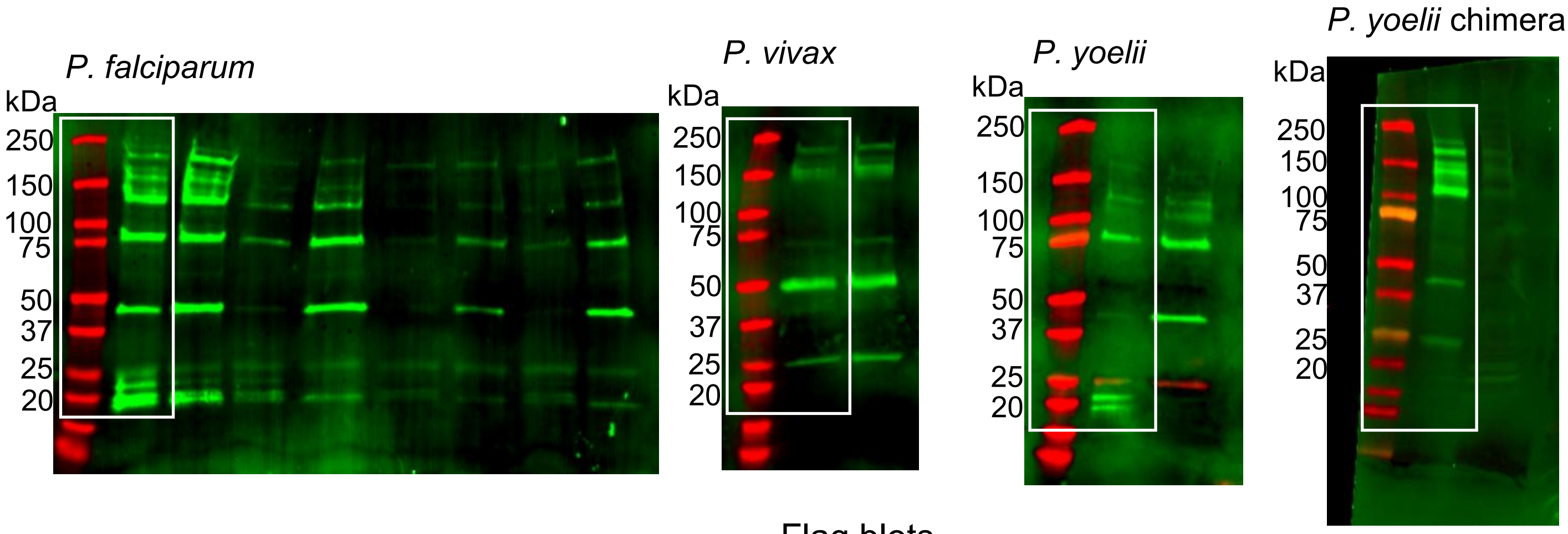

Flag blots
Supplementary Figure 4c

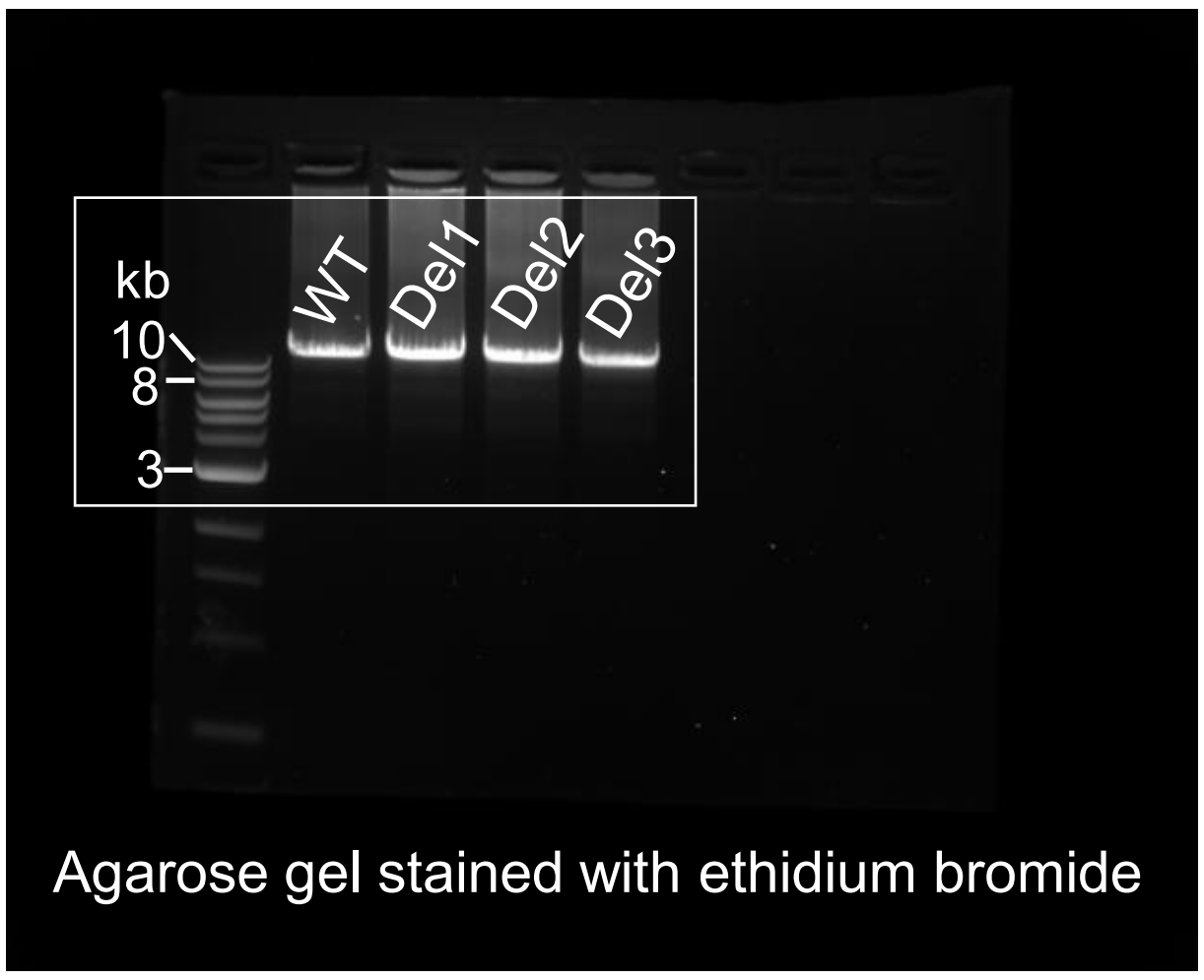

Supplementary Figure 5b

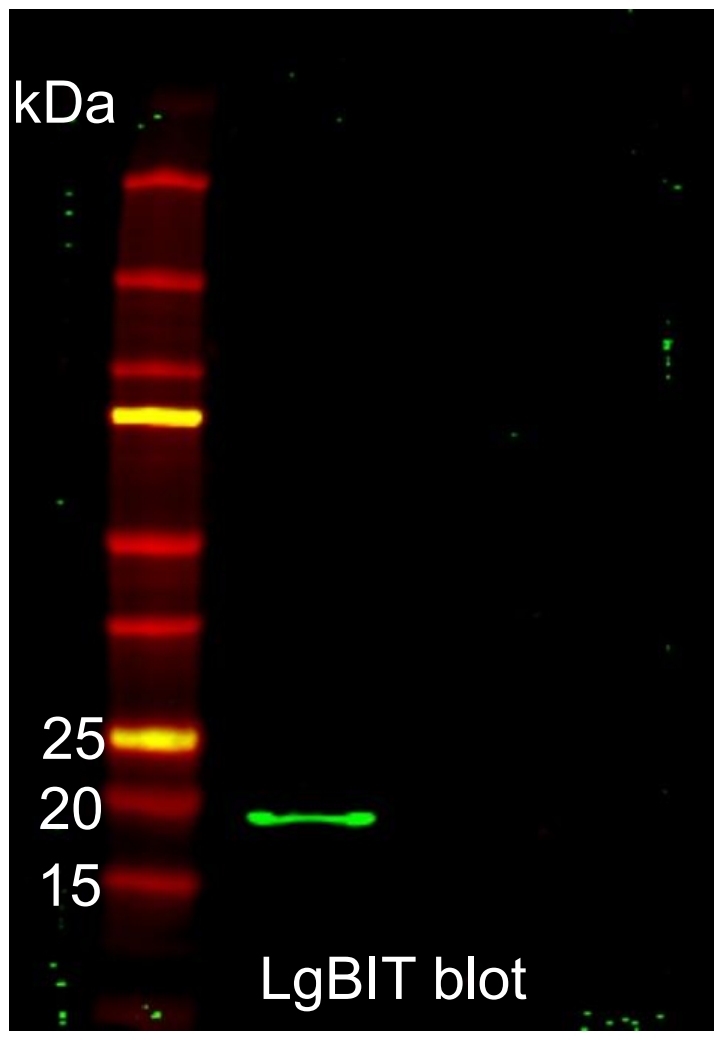

Supplementary Figure 6b

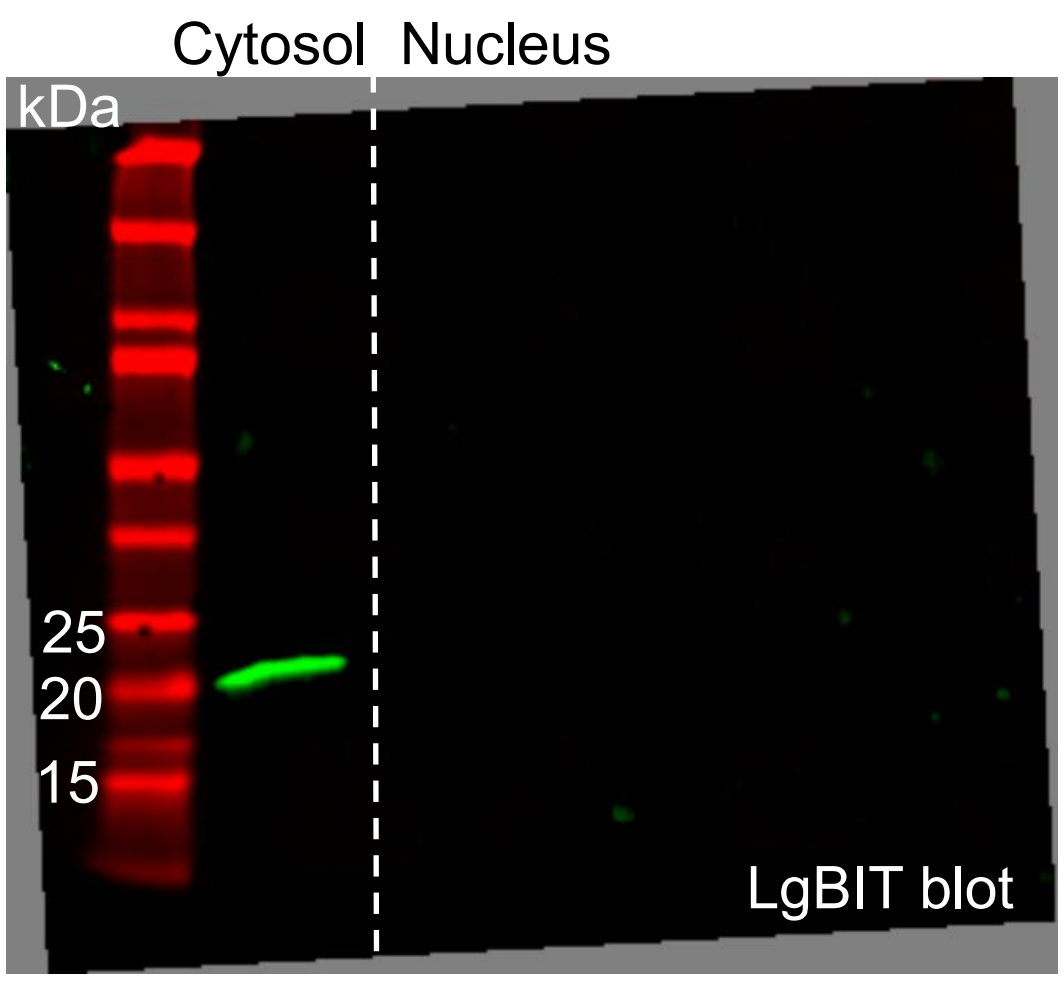

Supplementary Figure 6e

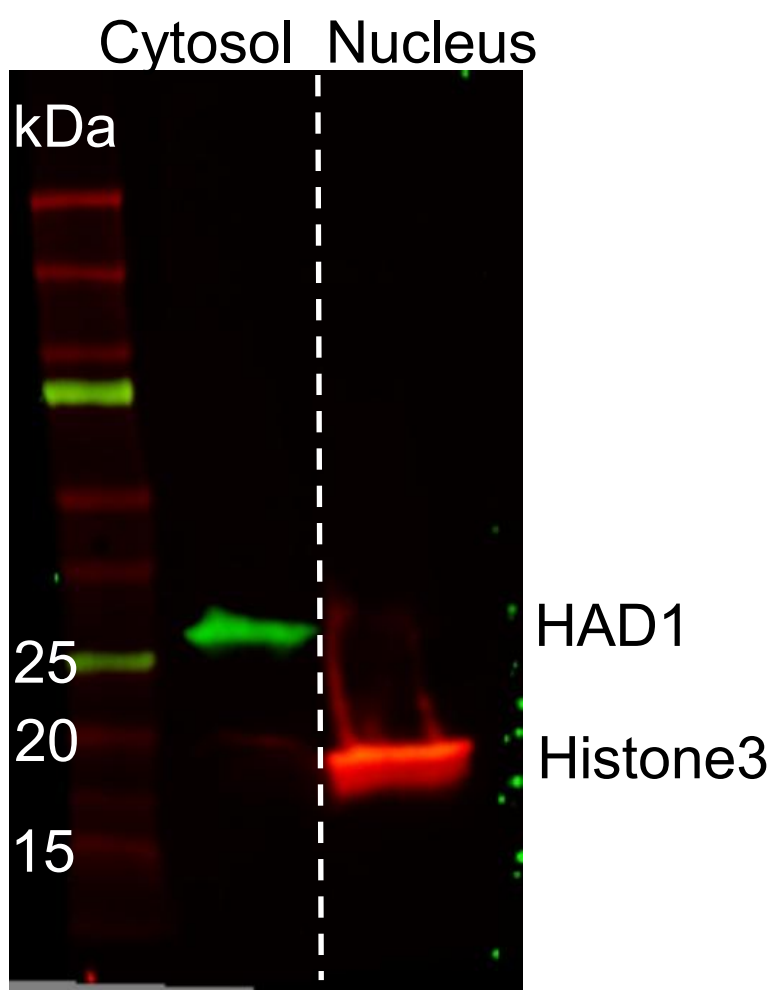

cytosolic and nuclear markers on
Supplementary Figure 6e
