# Supplementary material for: A role for the poly-asparagine repeat in the Plasmodium histone acetyltransferase, PfGCN5
Source: Nat Commun. 2026 Jun 20;17:7799. doi: 10.1038/s41467-026-74632-6 (PMC13438671; doi:10.1038/s41467-026-74632-6)
Supplement: Supplementary file 2 [file 41467_2026_74632_MOESM2_ESM.pdf]

Description of Additional supplementary files

Supplementary Data 1:

Complete list of proteins identified by mass spectrometry in PfGCN5 C-terminal pulldowns using anti-FLAG magnetic beads targeting the second copy. A summary of importance for this study is presented in Supplementary table 1.

Supplementary Data 2:

List of all the primers used in this study
