# Supplementary material for: A role for the poly-asparagine repeat in the Plasmodium histone acetyltransferase, PfGCN5
Source: Nat Commun. 2026 Jun 20;17:7799. doi: 10.1038/s41467-026-74632-6 (PMC13438671; doi:10.1038/s41467-026-74632-6)
Supplement: Supplementary file 3 [file 41467_2026_74632_MOESM5_ESM.pdf]

Corresponding author(s): Daniel E. Goldberg

Last updated by author(s): 06/04/2026

Reporting Summary

Nature Portfolio wishes to improve the reproducibility of the work that we publish. This form provides structure for consistency and transparency in reporting. For further information on Nature Portfolio policies, see our Editorial Policies and the Editorial Policy Checklist.

Statistics

For all statistical analyses, confirm that the following items are present in the figure legend, table legend, main text, or Methods section.

n/a	Confirmed	
☐	☒	The exact sample size (n) for each experimental group/condition, given as a discrete number and unit of measurement
☐	☒	A statement on whether measurements were taken from distinct samples or whether the same sample was measured repeatedly
☐	☒	The statistical test(s) used AND whether they are one- or two-sided<br>*Only common tests should be described solely by name; describe more complex techniques in the Methods section.*
☒	☐	A description of all covariates tested
☐	☒	A description of any assumptions or corrections, such as tests of normality and adjustment for multiple comparisons
☐	☒	A full description of the statistical parameters including central tendency (e.g. means) or other basic estimates (e.g. regression coefficient) AND variation (e.g. standard deviation) or associated estimates of uncertainty (e.g. confidence intervals)
☐	☒	For null hypothesis testing, the test statistic (e.g. F, t, r) with confidence intervals, effect sizes, degrees of freedom and P value noted<br>*Give P values as exact values whenever suitable.*
☒	☐	For Bayesian analysis, information on the choice of priors and Markov chain Monte Carlo settings
☒	☐	For hierarchical and complex designs, identification of the appropriate level for tests and full reporting of outcomes
☒	☐	Estimates of effect sizes (e.g. Cohen's d, Pearson's r), indicating how they were calculated

Our web collection on statistics for biologists contains articles on many of the points above.

Software and code

Policy information about availability of computer code

Data collection	n/a
Data analysis	Prism 11.0.0 was used to perform all analysis

For manuscripts utilizing custom algorithms or software that are central to the research but not yet described in published literature, software must be made available to editors and reviewers. We strongly encourage code deposition in a community repository (e.g. GitHub). See the Nature Portfolio guidelines for submitting code & software for further information.

Data

Policy information about availability of data

All manuscripts must include a data availability statement. This statement should provide the following information, where applicable:

- Accession codes, unique identifiers, or web links for publicly available datasets
- A description of any restrictions on data availability
- For clinical datasets or third party data, please ensure that the statement adheres to our policy

Authors confirm that all relevant data are included here and/or the supplementary information. Data used to generate each graph and uncropped gels or blots are provided as a Source Data file in a Microsoft Excel format. GCN5 sequence homology conservation across organisms and Plasmodium spp. are shown in Supplementary Fig. 1 and 2. Plasmid maps and cloning strategies are shown in Supplementary Fig. 3, 4, 5 and 6. Information for LC-MS/MS from C-terminal FLAG pull downs are summarized in Supplementary table 1. The entire list of pulled-down proteins can be found as Supplementary Data 1. Sequence coverage of PfGCN5

across deletion proteins is shown in Supplementary Fig.7. The Hhistone acetylation proteomic data generated in this study have been uploaded to MassIVE with identifiers MSV000099212 and PXD068464 [https://massive.ucsd.edu/ProteoSAFe/dataset.jsp?task=aad3332d2158402bb4a7d10724dae948]. The list of primers used in this study is presented asin Supplementary table Data 2. Source data are provided with this paper.

Research involving human participants, their data, or biological material

Policy information about studies with human participants or human data. See also policy information about sex, gender (identity/presentation), and sexual orientation and race, ethnicity and racism.

Reporting on sex and gender	n/a
Reporting on race, ethnicity, or other socially relevant groupings	n/a
Population characteristics	n/a
Recruitment	n/a
Ethics oversight	n/a

Note that full information on the approval of the study protocol must also be provided in the manuscript.

Field-specific reporting

Please select the one below that is the best fit for your research. If you are not sure, read the appropriate sections before making your selection.

☒ Life sciences ☐ Behavioural & social sciences ☐ Ecological, evolutionary & environmental sciences

For a reference copy of the document with all sections, see nature.com/documents/nr-reporting-summary-flat.pdf

Life sciences study design

All studies must disclose on these points even when the disclosure is negative.

Sample size	No sample-size calculation was performed. Three or four biological replicates in technical triplicate/quadruplicate were performed to ensure reproducibility.
Data exclusions	No data was excluded from analysis.
Replication	Each biological replicate contained 3-4 technical replicates. In every case, replication was successful.
Randomization	Not relevant for this study. It was necessary to ensure no mixing between experimental groups for the analysis of the data.
Blinding	Not relevant for this study. The outcomes were not subjective to the knowledge of the researcher i. e. pain

Reporting for specific materials, systems and methods

We require information from authors about some types of materials, experimental systems and methods used in many studies. Here, indicate whether each material, system or method listed is relevant to your study. If you are not sure if a list item applies to your research, read the appropriate section before selecting a response.

Materials & experimental systems

n/a	Involved in the study	
☐	☒	Antibodies
☐	☒	Eukaryotic cell lines
☒	☐	Palaeontology and archaeology
☒	☐	Animals and other organisms
☒	☐	Clinical data
☒	☐	Dual use research of concern
☒	☐	Plants

Methods

n/a	Involved in the study	
☒	☐	ChIP-seq
☒	☐	Flow cytometry
☒	☐	MRI-based neuroimaging

Antibodies

Antibodies used

Rabbit anti-HA 1:1,000 (clone: polyclonal, provider: Sigma, catalog#: H6908, Lot #: 0000097652)
Rat anti-FLAG 1:1,000 (clone: L5, provider: Biolegend, catalog#: 637301, Lot#: B364917)
Mouse anti-Histone H3 1:1000 (clone: 1G1, provider: EPIGENTEK, catalog#: A68386, Lot#: 2410006)
Mouse anti-H3K9ac 1:1,000 (clone: 1B10, provider: Active Motif, catalog#: 61952, Lot#: 22305107-14)
Mouse anti-LgBiT 1:250 (monoclonal, provider: Promega, catalog#: N710A, Lot#: 0000609287)
Mouse anti-HiBiT 1:500 (monoclonal, provider: Promega, catalog#: N720A, Lot#: 0000567545)
Rabbit anti-Plasmodium aldolase 1:1,000 (polyclonal, provider: abcam, catalog#: ab38905, Lot#: 000078906)
Rabbit anti-HAD1 1:1,000 was provided by Dr. Audrey Odom at University of Pennsylvania
Mouse anti-PMV 1:500 was developed by the Hybridoma Center at WashU

Validation

-Rabbit anti-HA: Anti-HA: produced in rabbit using a synthetic peptide corresponding to amino acid residues 98-106 (Tyr-ProTyr-Asp-Val-Pro-Asp-Tyr-Ala) of the human Influenza hemagglutinin (HA), conjugated to KLH. The antibody is affinity-purified on the immobilized immunizing peptide (https://www.sigmaaldrich.com/US/en/product/sigma/h6908?srsltid=AfmBOoqW3IXpXltB4cl-CjGxXhOQLj62aMHQtG3KGnEufwy4K1wX8UtP). Validated in P. falciparum in Mukherjee, S., Nguyen, S., Sharma, E. et al. Maturation and substrate processing topography of the Plasmodium falciparum invasion/egress protease plasmepsin X. Nat Commun 13, 4537 (2022). https://doi.org/10.1038/s41467-022-32271-7.
-Rat anti-FLAG: Cell extracts from untransfected 293T cells or 293T cells transfected with a plasmid encoding DYKDDDDK(FLAG)-tagged protein were screened using anti-(FLAG)DYKDDDDK, clone L5 in a western blot. The antibody specifically recognized lysates from transfected cells but no untransfected (https://www.biolegend.com/nl-nl/products/purified-anti-dykddddk-tag-antibody-4905). Validated in P. falciparum in this manuscript: endogenous PfGCN5-GFP-HA and second copy PfGCN5-FLAG exhibit the same processing pattern.
-Mouse anti-Histone H3: recombinant protein raised in mouse. Unconjugated with specificity to a wide range of orgnanisms including human, mouse, rat, yeast (https://www.epigentek.com/catalog/histone-h3-monoclonal-antibody-1g1-p-60181.html?srsltid=AfmBOoqlpDO8ccYPc611B5lfVhHD9sjVBPkH6h1idcxoTzhbTwuyvply). Validated in P. falciparum in this study: A band of ~17 kDa was observed and its abundance was stage dependent being highly expressed in schizont stages and lowly expressed in ring stages.
-Mouse anti-H3K9ac: monoclonal antibody (IgG3 isotype), purified by protein A chromatography and raised against a peptide including acetyl-lysine 9 of human Histone H3 with reactivity to a wide range of organisms (https://www.google.com/search?q=Active+Motif%2C+catalog%23%3A+61952&oq=Active+Motif%2C+catalog%23%3A+61952&gs_lcrp=EgZjaHJvbWUqBggAEEUYOzIGCAAQRRg7MgcIARAhGI8CMgcIAhAhGI8C0gEIMzQ0MGowajSoAgCwAgA&sourceid=chrome&ie=UTF-8). Validated in P. falciparum in this study: a band of ~17kDa with distinct abundance from that seen from unmodified histone H3 was detected.
-Mouse anti-LgBiT : protein A/G affinity-purified mouse monoclonal antibody that is used to detect Large BiT (LgBiT) and LgBiT fusion proteins via Western blotting and immunofluorescence (https://www.promega.com/products/protein-detection/primary-and-secondary-antibodies/anti-lgbit-monoclonal-antibody/?catNum=N7100). Validated in P. falciparum in this study: this antibody detected a protein of 18 kDa (expected size). As in other systems this protein was found to be exclusively cytosolic in P. falciparum and binding to PfGCN5-HiBiT was readily detected.
-Mouse anti-HiBiT: Anti-HiBiT Monoclonal Antibody(a) is a potent and specific mouse monoclonal antibody (mAb) that binds to the HiBiT tag, enabling applications such as immunoblotting, immunofluorescence, immunoprecipitation and fluorescence-activated cell sorting (FACS). The antibody binds to HiBiT with high affinity, with a KD in solution of approximately 6pM. Because of this high affinity and low background binding, many HiBiT-tagged proteins can be detected at endogenous expression levels. Raised in mouse and purified with protein A resin. Unconjugated monoclonal antibody (https://www.promega.com/products/protein-detection/protein-quantification/anti-hibit-antibody/?catNum=N7200). Validated in P. falciparum in this study: PfGCN5-HiBiT protein exhibited an N-terminal processing pattern previously reported in Rawat et al. Scietific Reports, 2021. https://doi.org/10.1038/s41598-020-79539-w
-Rabbit anti-Plasmodium aldolase: Rabbit Polyclonal ALF antibody - conjugated to HRP. Suitable for ELISA, WB and reacts with Plasmodium falciparum samples (https://www.abcam.com/en-us/products/primary-antibodies/hrp-plasmodium-aldolase-antibody-ab38905). Cited in Gomes et al. Nature, 2022. DOI: 10.1038/s41586-022-05509-z
-Rabbit anit-HAD1 was provided by Dr. Audrey Odom and validated in Guggisberg A. M. et al. Nature communications, 2014. https://doi.org/10.1038/ncomms5467
-Mouse anti-PMV was develop by the Hybridoma Center at WashU and validated in Banerjee R. et al. PNAS, 2002. https://doi.org/10.1073/pnas.022630099

Eukaryotic cell lines

Policy information about cell lines and Sex and Gender in Research

Cell line source(s)

We used the NF54 attB DiCre line which was made by Dr. Josh Beck at IOWA State University and described in Fierro M. A. et al. PNAS, 2023. DOI: 10.1073/pnas.2308676120

Authentication

Whole-genome sequencing

Mycoplasma contamination

Negative

Commonly misidentified lines
(See ICLAC register)

n/a

Plants

Seed stocks	n/a
Novel plant genotypes	n/a
Authentication	n/a
