# Supplementary material for: A role for the poly-asparagine repeat in the Plasmodium histone acetyltransferase, PfGCN5
Source: Nat Commun. 2026 Jun 20;17:7799. doi: 10.1038/s41467-026-74632-6 (PMC13438671; doi:10.1038/s41467-026-74632-6)
Supplement: Supplementary file 4 [file 41467_2026_74632_MOESM6_ESM.pdf]

nature portfolio

Peer Review File

A role for the poly-asparagine repeat in the Plasmodium histone acetyltransferase, PfGCN5

Corresponding Author: Professor Daniel Goldberg

This file contains all reviewer reports in order by version, followed by all author rebuttals in order by version.

Version 0:

Reviewer comments:

Reviewer #1

(Remarks to the Author)
In this study, the authors used poly-N repeats in the N-terminal of PfGCN5 as an example to explore the potential function of N repeats, a unique feature in the malaria parasite Plasmodium falciparum. N repeats-deletion in PfGCN5 resulted in defects in asexual parasite growth, being more sensitive to heat shock stress conditions, and a reduction in histone acetylation. The authors further found that PfGCN5 was processed in the nucleus, inconsistent with recent findings that PfGCN5 is processed in the food vacuole. Lastly, the authors claimed that N-repeats conferred stability of the N-terminal polypeptide and interacted with the C-terminal domain by using the LgBiT-HiBiT system.
Overall, this study has significant findings on the function of poly-N-repeat in the malaria parasite. However, some conclusions are not clear-cut and need more evidence.
1. The authors claimed that N-repeats confers stability of N-terminal polypeptide based on Western blots of HiBiT-tagged Del3-PfGCN5, showing no bands was recognized by anti-HiBiT, indicating N-terminal polypeptide is degraded (Fig. 8B). HiBiT tag was inserted at about 150 aa of N-terminal of PfGCN5 (Fig. 1A). If HiBiT tag was choped out from N-terminal, that means the sized of truncated PfGCN5 will be at least 18kDa smaller than the regular PfGCN5 processed bands, however, Western blots in Fig. 3d did not show any size differences between WT and C-termial FLAG tagged Del3-PfGCN5. Additionally, the reason affinity purification of Del1-Del3-PfGCN5-associated proteins did not find N-terminal peptides could be that the N-repeats deletions cause a weaker association between N-terminal fragments and C-terminal fragments.
2. The authors claimed that N-repeat interacts with the C-terminal domain by using an in vitro association assay (Fig. 8G). The authors showed the results from the pull-down of C-terminal FLAG-tagged fragments and blotted for N-terminal HiBit-tagged fragments. The 120KDa HiBit-tagged fragment is supposed to be processed from full-length PfGCN5 (190kDa) into a 70KDa FLAG-tagged fragment. How about 110kDa HiBit-tagged fragment?
3. Deletion of N-repeat (Del3-PfGCN5) led to a reduction of histone acetylation. This is not the direct evidence showing HAT activity is reduced after deletion. The authors can purify WT-PfGCN5 and Del3-PfGCN5 from parasites and compare HAT activities. Interestingly, H4K8acK16ac were also reduced upon PfGCN5 KO and Del3. It is known that H4 acetylation is catalyzed by MYST, thus, this change could be indirectly caused by PfGCN5. Additionally, a more detailed description of the changes in histone modification is needed, besides H3K9ac.
4. Authors showed a lot of Western blots for PfGCN5 expression (Fig. 2C, 3d, 7a, 7c, 8a, 8d, 8g). The patterns of those WBs were not the same (always changing). Could this be because of stage differences? Or others?
5. Not sure if affinity purification of PfGCN5-associated proteins also identified AP2-LT besides PHD1, PHD2, ADA2, and NAPS? Yeast-two hybrid study showed that PfGCN5 N-terminal (aa716-aa964) binds AP2-LT. Deletion of N-repeats may cause a structure change of the N-terminal fragment, and weaken the association to AP2-LT, which eventually impacts GCN5 HAT activity.

Reviewer #2

(Remarks to the Author)

The manuscript aims to investigate the functional significance of the poly-asparagine (N) repeat located within the N-terminal region of the P. falciparum histone acetyltransferase PfGCN5, an essential protein involved in epigenetic regulation. Using a combination of genetic editing, immunoblotting, proteomics, and microscopy approaches, the authors present a set

of experiments aimed at elucidating how this repetitive domain contributes to parasite development, chromatin organization, and stress response.
The manuscript is well written, and the figures are clear and largely support the conclusions. The results provide very interesting insights on the putative function of poly-asparagine repeats in P. falciparum and their possible involvement in proper protein function. It will thus add valuable new insights to the field of molecular biology/biochemistry in P. falciparum.
While the study presents a substantial amount of experimental work, several aspects of the methodology, should be revisited before publication.

Major comments:
1. Generation of parasite lines: the manuscript lacks details on the cloning strategies and plasmids used to create the rescue, deletion, and LgBiT-HiBiT parasite lines. Furthermore, no data is provided to verify the correct integration of the constructs at the targeted gene loci. To better understand how the cell lines were generated, the authors should include the plasmids design and integration strategies along with evidence of the correct integration of the plasmids (e.g. by PCR of gDNA).
2. Design of the chimera rescue construct: In figure 2D, the authors show the schematic representation of two plasmodial GCN5 homologous and a chimera construct to rescue the PfGCN5 KO. However, the chimera and the native P. falciparum constructs are similar making it hard to interpret and compare the phenotype from these two parasite lines. In my view the experiment would be much more conclusive if the parasite line would constitute an actual domain swap, meaning only the insertion of the poly-asparagine domain of Pf into the Py construct, to really pinpoint the rescue function on that domain and exclude other confounding effects from the rest of the Pf N-term.

Minor Comments

1. Immunofluorescence assays pictures:The immunofluorescence images appear to have undergone processing, but the manuscript does not specify which software or image-processing settings were used. The authors should clearly describe the image acquisition parameters, processing workflow, and software employed to obtain the final images.

2. Typographical error (line 223): There appears to be a typographical error—a question mark enclosed in parentheses—that should be corrected.

3. Figure 5a labeling: In Figure 5a, there appears to be a labeling error for the time point “24–47,” which should be corrected for consistency.

4. PfGCN5 localization and trafficking (Figure 7a/b): In Figure 7a/b, the authors present Western blots and immunofluorescence data using the HA tag to detect PfGCN5 and conclude that the protein localizes exclusively to the nucleus. However, this observation contrasts with previous findings (Bhowmick et al., 2021). To strengthen this conclusion, it would be advisable to include a cytoplasmic marker in the IFA (as in Figure 3a) and perform Western blotting on fractionated samples. These additional controls could help asess the proposed hypothesis of an ER–Golgi–independent trafficking pathway.

Version 1:

Reviewer comments:

Reviewer #1

(Remarks to the Author)
The authors succefully addressed my concerns and comments.

Reviewer #2

(Remarks to the Author)
The revised manuscript represents a substantial improvement over the original submission, with more detailed methodological descriptions and an expanded discussion of the results.
The authors have adequately addressed the major and minor concerns raised in the previous review and have incorporated additional experimental evidence and new figures that strengthen the quality of the study. In summary, all previous comments have been satisfactorily addressed.

Reviewer #3

(Remarks to the Author)
I co-reviewed this manuscript with one of the reviewers who provided the listed reports. This is part of the Nature Communications initiative to facilitate training in peer review and to provide appropriate recognition for Early Career Researchers who co-review manuscripts.

Open Access This Peer Review File is licensed under a Creative Commons Attribution 4.0 International License, which permits use, sharing, adaptation, distribution and reproduction in any medium or format, as long as you give appropriate credit to the original author(s) and the source, provide a link to the Creative Commons license, and indicate if changes were made.
In cases where reviewers are anonymous, credit should be given to 'Anonymous Referee' and the source.
The images or other third party material in this Peer Review File are included in the article's Creative Commons license, unless indicated otherwise in a credit line to the material. If material is not included in the article's Creative Commons license and your intended use is not permitted by statutory regulation or exceeds the permitted use, you will need to obtain permission directly from the copyright holder.
To view a copy of this license, visit https://creativecommons.org/licenses/by/4.0/

We thank the reviewers for their constructive comments and have revised the manuscript to clarify ambiguities and to add the two requested experiments.

REVIEWER COMMENTS

Reviewer #1 (Remarks to the Author):

In this study, the authors used poly-N repeats in the N-terminal of PfGCN5 as an example to explore the potential function of N repeats, a unique feature in the malaria parasite Plasmodium falciparum. N repeats-deletion in PfGCN5 resulted in defects in asexual parasite growth, being more sensitive to heat shock stress conditions, and a reduction in histone acetylation. The authors further found that PfGCN5 was processed in the nucleus, inconsistent with recent findings that PfGCN5 is processed in the food vacuole. Lastly, the authors claimed that N-repeats conferred stability of the N-terminal polypeptide and interacted with the C-terminal domain by using the LgBiT-HiBiT system. Overall, this study has significant findings on the function of poly-N-repeat in the malaria parasite. However, some conclusions are not clear-cut and need more evidence.

1. The authors claimed that N-repeats confers stability of N-terminal polypeptide based on Western blots of HiBiT-tagged Del3-PfGCN5, showing no bands was recognized by anti-HiBiT, indicating N-terminal polypeptide is degraded (Fig. 9B). HiBiT tag was inserted at about 150 aa of N-terminal of PfGCN5 (Fig. 1A). If HiBiT tag was choped out from N-terminal, that means the sized of truncated PfGCN5 will be at least 18kDa smaller than the regular PfGCN5 processed bands, however, Western blots in Fig. 3d did not show any size differences between WT and C-terminal FLAG tagged Del3-PfGCN5.
Additionally, the reason affinity purification of Del1-Del3-PfGCN5-associated proteins did not find N-terminal peptides could be that the N-repeats deletions cause a weaker association between N-terminal fragments and C-terminal fragments.

The longest C-terminal fragment runs at about 120 kDa, corresponding to approximately 1,100 amino acids, which suggests a cleavage site around amino acid position 366. The poly-N repeat is positioned between residues 143 and 240. Thus, none of the C-terminal fragments should contain the poly-N repeat, and the blot in Fig. 3d confirms this observation. While deleting the poly-N repeat may have resulted in a weaker association between N- and C-terminal fragments, HiBiT blots of the Del3-HiBiT line indicate that the N-terminal polypeptide is degraded (Fig. 9b), and mass spectrometry failed to detect N-terminal fragments in C-terminal pulldowns (Supplementary Fig 7).

Therefore, the association between N- and C-terminal fragments is not seen in the deletion line.

2. The authors claimed that N-repeat interacts with the C-terminal domain by using an in vitro association assay (Fig. 9G). The authors showed the results from the pull-down of C-terminal FLAG-tagged fragments and blotted for N-terminal HiBit-tagged fragments. The 120KDa HiBit-tagged fragment is supposed to be processed from full-length PfGCN5 (190kDa) into a 70KDa FLAG-tagged fragment. How about 110kDa HiBit-tagged fragment?

The processing of this protein is complex. However, from our N- and C-terminal blots, we can say that the 120 kDa fragments we see in both N- and C-terminal blots are not the same; the 120 kDa N-terminally HiBiT-tagged fragment does not contain the FLAG-tagged C-terminus. This is because mapping ~1,100 amino acids corresponding to a 120 kDa fragment from either the N- or C-terminus does not encompass the opposite tagged-end. Having clarified that, our observations support an alternative cleavage process in which a full-length molecule can be processed into an N- or C-terminal 120 kDa fragment. Processing of the full-length protein into an N-terminal HiBiT-tagged fragment of 120 kDa generates a C-terminal FLAG-tagged fragment of 70 kDa. Further processing of the N-terminal 120 kDa fragment into a 110 kDa HiBiT-tagged piece produces an 80 kDa C-terminal FLAG-tagged fragment. In all C-terminal blots, we have labelled the 70 and 80 kDa fragments seen as a doublet just above the 100 kDa ladder mark given the presence of GFP in the C-terminus. We have also enumerated the c-terminal fragments in Supplementary Fig. 3d and in the main text on page 7 to help clarify. Finally, we have mentioned the alternative cleavages in the 5th paragraph of the discussion.

3. Deletion of N-repeat (Del3-PfGCN5) led to a reduction of histone acetylation. This is not the direct evidence showing HAT activity is reduced after deletion. The authors can purify WT-PfGCN5 and Del3-PfGCN5 from parasites and compare HAT activities. Interestingly, H4K8acK16ac were also reduced upon PfGCN5 KO and Del3. It is known that H4 acetylation is catalyzed by MYST, thus, this change could be indirectly caused by PfGCN5. Additionally, a more detailed description of the changes in histone modification is needed, besides H3K9ac.

We thank the reviewer for suggesting that we measure histone acetylation *in vitro*. We have performed this experiment and found that both the PfGCN5 WT and deletion protein are equally active (New Fig. 7 and new results section). We also added a new paragraph to the end of the discussion to entertain possible mechanisms of indirect action.

We have also provided a detailed description of all the different histone modifications affected by the removal of the poly-N repeat *in vivo* in the discussion section.

4. Authors showed a lot of Western blots for PfGCN5 expression (Fig. 2C, 3d, 8a, 8c, 9a, 9d, 9g). The patterns of those WBs were not the same (always changing). Could this be because of stage differences? Or others?

The only differences attributable to staging exist between 3-24hpi (Supplementary Fig. 3d), where most of the full-length protein is processed into 120, 80, 70, 40, 20, and 18 kDa forms. Except for the western blot on Supplementary Fig. 3d, all blots were made with lysates from parasites older than 24hpi. The endogenous protein was C-terminally tagged with GFP-3HA (Fig. 2c, 8a, 8c), giving ~30 kDa extra to the mass of each band in 3HA blots, in comparison to FLAG blots, which detect the second copy C-terminally tagged with FLAG (Fig. 3d, 7a, 9g). Additionally, the second copy was N-terminally tagged with HiBiT, resulting in a distinct band pattern from that observed in C-terminal blots (Fig. 9a, 9d). We appreciate the reviewer's comment and have added additional labelling to all C-terminal 3HA blots, indicating the mass of each band after subtracting that of GFP, so that the reader can differentiate between blots illustrating the endogenous or the second copy proteins C-terminally tagged.

5. Not sure if affinity purification of PfGCN5-associated proteins also identified AP2-LT besides PHD1, PHD2, ADA2, and NAPS? Yeast-two hybrid study showed that PfGCN5 N-terminal (aa716-aa964) binds AP2-LT. Deletion of N-repeats may cause a structure change of the N-terminal fragment, and weaken the association to AP2-LT, which eventually impacts GCN5 HAT activity.

C-terminal pull-downs identified AP2-I (PF3D7_1007700) and a putative AP2 protein (PF3D7_1107800), but AP2-LT (PF3D7_0802100) was not retrieved. In pilot trials that we did not report here, AP2-LT was pulled down with low abundance. Therefore, it is possible that our buffer conditions did not favor AP2-LT interaction with PfGCN5.

Reviewer #2 (Remarks to the Author):

The manuscript aims to investigate the functional significance of the poly-asparagine (N) repeat located within the N-terminal region of the P. falciparum histone acetyltransferase PfGCN5, an essential protein involved in epigenetic regulation. Using a combination of genetic editing, immunoblotting, proteomics, and microscopy approaches, the authors present a set of experiments aimed at elucidating how this

repetitive domain contributes to parasite development, chromatin organization, and stress response.

The manuscript is well written, and the figures are clear and largely support the conclusions. The results provide very interesting insights on the putative function of poly-asparagine repeats in P. falciparum and their possible involvement in proper protein function. It will thus add valuable new insights to the field of molecular biology/biochemistry in P. falciparum. While the study presents a substantial amount of experimental work, several aspects of the methodology, should be revisited before publication.

Major comments:

1. Generation of parasite lines: the manuscript lacks details on the cloning strategies and plasmids used to create the rescue, deletion, and LgBiT-HiBiT parasite lines. Furthermore, no data is provided to verify the correct integration of the constructs at the targeted gene loci. To better understand how the cell lines were generated, the authors should include the plasmids design and integration strategies along with evidence of the correct integration of the plasmids (e.g. by PCR of gDNA).

We appreciate these suggestions and have included plasmid maps, evidence of correct genetic integration, and expanded details about our cloning strategies for each line. Readers can find this information in the Supplementary Information file. We have also made the nomenclature more consistent in the text for clarity.

2. Design of the chimera rescue construct: In figure 2D, the authors show the schematic representation of two plasmodial GCN5 homologous and a chimera construct to rescue the PfGCN5 KO. However, the chimera and the native P. falciparum constructs are similar making it hard to interpret and compare the phenotype from these two parasite lines. In my view the experiment would be much more conclusive if the parasite line would constitute an actual domain swap, meaning only the insertion of the poly-asparagine domain of Pf into the Py construct, to really pinpoint the rescue function on that domain and exclude other confounding effects from the rest of the Pf N-term.

We agree with the reviewer that swapping the poly-N repeat into the *P. yoelii* GCN5 would be a cleaner design. However, because there is so little homology in the N-terminus of GCN5 across *Plasmodium* species, we could not determine or make an informed guess of an adequate place to insert the repeat in the *P. yoelii* GCN5 coding sequence. Given this constraint, we decided to swap a larger N-terminal region, whose length was determined by homology. In this way, we also hoped not to alter protein processing, localization, or function.

Minor Comments

1. Immunofluorescence assays pictures:The immunofluorescence images appear to have undergone processing, but the manuscript does not specify which software or image-processing settings were used. The authors should clearly describe the image acquisition parameters, processing workflow, and software employed to obtain the final images.

Thank you for pointing out this omission. We have added the following sentences to the Materials and Methods section under the subtitle Immunofluorescence assays: “Images were taken in a Zeiss AxioImager M1 epifluorescence microscope with a Hamamatsu ORCA-ER CCD camera and the AxioVision software v. 4.8.1 using a 100x objective. Scale bars, and adjustments to brightness and contrast were made for display purposes with Zen Lite v. 3.12 (Zeiss).”

2. Typographical error (line 223): There appears to be a typographical error—a question mark enclosed in parentheses—that should be corrected.

Thank you for catching this typo. We have corrected it.

3. Figure 5a labeling: In Figure 5a, there appears to be a labeling error for the time point “24–47,” which should be corrected for consistency.

Thank you for catching this typo. We have corrected it.

4. PfGCN5 localization and trafficking (Figure 8a/b): In Figure 8a/b, the authors present Western blots and immunofluorescence data using the HA tag to detect PfGCN5 and conclude that the protein localizes exclusively to the nucleus. However, this observation contrasts with previous findings (Bhowmick et al., 2021). To strengthen this conclusion, it would be advisable to include a cytoplasmic marker in the IFA (as in Figure 3a) and perform Western blotting on fractionated samples. These additional controls could help asess the proposed hypothesis of an ER–Golgi–independent trafficking pathway.

The fractionation experiment has been added as Fig. 8c. There is a clear accumulation of full-length protein in the nucleus upon E64d treatment.
